# Reducing effects of dispersal on the bias of 2-sample mark-recapture estimators of stream fish abundance

**DOI:** 10.1371/journal.pone.0200733

**Published:** 2018-08-01

**Authors:** James N. McNair, Carl R. Ruetz, Ariana Carlson, Jiyeon Suh

**Affiliations:** 1 Annis Water Resources Institute, Grand Valley State University, Muskegon, Michigan, United States of America; 2 Department of Statistics, Grand Valley State University, Allendale, Michigan, United States of America; 3 Department of Mathematics, Grand Valley State University, Allendale, Michigan, United States of America; Université du Québec à Trois-Rivières, CANADA

## Abstract

The 2-sample mark-recapture method with Chapman’s estimator is often used by inland fishery managers to estimate the reach-scale abundance of stream fish. An important assumption of this method is that no dispersal into or out of the study reach occurs between the two samples. Violations of this assumption are probably common in practice, but their effect on bias (systematic error) of abundance estimates is poorly understood, especially in small populations. Estimation methods permitting dispersal exist but, for logistical reasons, often are infeasible for routine assessments in streams. The purpose of this paper is to extend available results regarding effects of dispersal on the bias of Chapman’s estimator as applied to reach-scale studies of stream fish abundance. We examine for the first time the joint effects of dispersal and sampling variation on the bias of this estimator. To reduce the bias effects of dispersal, we propose a modified sampling scheme in which the original study reach is expanded, a central subreach is sampled during the mark session (sample 1), and the entire reach is sampled during the recapture session (sample 2). This modified sampling scheme can substantially reduce bias effects of dispersal without requiring unique marking of individual fish or additional site visits. Analytical and simulation results show that sampling variation tends to create negative bias with respect to study-reach abundance, while dispersal tends to create positive bias; the net effect can be positive, negative, or zero, depending on the true abundance, capture probabilities, and amount and nature of dispersal. In most cases, simply expanding the study reach is an effective way to reduce dispersal-related bias of Chapman’s estimator, but expanding the study reach and employing the modified sampling scheme we propose is a better alternative for accurately estimating abundance with the same level of sampling effort.

## Introduction

Estimating abundance is an important component of conservation and natural resource management studies that deal with issues such as population viability or the status of harvested populations. In most cases, it is not feasible to count every individual, so any attempt at a direct census would underestimate abundance. Various sampling methods and statistical estimators have therefore been devised that can produce accurate abundance estimates based on partial counts of a population, provided certain method-specific assumptions are satisfied [1–4].

One of the simplest methods of abundance estimation is the 2-sample mark-recapture (capture-recapture) method, which has been used to estimate abundance in marine and freshwater fisheries since 1904 or earlier [5]. An initial sample is taken from the study area and the captured individuals are counted, marked, and released. Later, a second sample is taken and the numbers of marked and unmarked individuals are determined. From these data, abundance in the study area can be estimated (see below). The main assumptions are that the same individuals are present for both samples (no recruitment, mortality, or dispersal across the study-area boundary occurs between samples), all individuals have the same capture probability during a given sampling session, all marks are retained between samples, and all recaptured individuals are recognized as marked [1, 3, 4].

Despite the availability of more-sophisticated alternatives, the 2-sample mark-recapture method remains one of the most commonly used methods for estimating reach-scale abundance of fish in streams around the world [6–17]. Stream fisheries are often managed at the reach (subpopulation) scale, requiring governmental agencies responsible for managing inland fisheries to estimate abundance at large numbers of sites across the many stream reaches within their jurisdictional boundaries. These estimates are used for a variety of management purposes, such as documenting temporal trends to assess the efficacy of fishing regulations and comparing abundance among reaches of a stream to document the spatial distribution of fish and assess the need for reach-specific habitat enhancement. Properties of the 2-sample mark-recapture method that are especially advantageous in this context are that it requires only temporary batch marking (usually with partial fin clips, which are quick and require no tag purchases) and only two site visits (usually on consecutive days), yet it produces accurate abundance estimates when its assumptions are appropriate and at least 7 recaptures are made [11, 12, 18].

The history of the 2-sample mark-recapture method is detailed in a thorough review by Goudie and Goudie [5], who correct several historical myths that are prevalent in the literature on abundance estimation. They demonstrate that using recaptures of marked fish to estimate population size was already an established practice among northern European fisheries scientists by 1904 when Meek [19] reported such an estimate for English plaice and noted that the method had been used before [5]. Such estimates appear in various European fisheries reports and publications after 1904 (e.g., Garstang [20] in 1905), most notably the book and papers by the Norwegian fisheries scientist Dahl published between 1917 and 1919 [5, 21–23]. This methodology was eventually adopted by investigators studying other organisms, including Pearse in 1923 (North American turtles) [24], H.D. and E. B. Ford in 1930 (British butterflies) [25], and Lincoln in 1930 (North American waterfowl) [26].

All of these early abundance estimates employed the simple ratio estimator

$$\hat{N}=c^{\prime }m/r^{\prime },$$ (1)

where $\hat{N}$ is estimated abundance, *m* is the number of individuals caught, marked, and released in the first sample, *c*′ is the total number of individuals caught in the second sample, and *r*′ is the number of marked individuals in the second sample (throughout this paper, a prime (′) denotes quantities specific to the second sample). Dahl published the first explicit derivation of this estimator in 1917 (in Norwegian) [21] and 1919 (in English) [23], lucidly explaining the underlying rationale. From his 1919 paper:

“If I have marked 100 trout and distributed them evenly, and if I then fish and capture 150 trout, of which 50 prove to have been marked, I have taken half of the marked fish. Considering the marked fish as representative of the stock I should consequently have taken half of the stock of the tarn and the total number contained should be about 300 fish. In other words, if I divide the number of marked fish liberated [*m* in Eq (1)] by the number of marked fish recaptured [*r*′], I obtain a coefficient of capture, and by multiplying the total number or weight of fish taken [*c*′] with this coefficient, I obtain an approximately correct estimate of the total number and weight of fish present in the water.”

Dahl also appears to have been the first to apply this estimator to data (seine samples from small tarns) for which the basic assumptions were plausible and the value of *c*′ was accurately known [5].

The estimator in Eq (1) is referred to by a variety of eponyms in the literature—Petersen, Petersen-Lincoln, Lincoln-Petersen, and Lincoln estimator or index—all of which are historically erroneous (egregiously so in the case of Lincoln, who merely popularized application of the estimator to North American waterfowl) and therefore will be avoided here. Goudie and Goudie [5] show that this estimator emerged communally among fisheries scientists in northern Europe circa 1900, so it seems both pointless and unjust to attempt to associate a particular individual’s name with it. We therefore will call it the MRR (mark-recapture ratio) estimator, with *m*/*r*′ in Eq (1) being the mark-recapture ratio and *c*′ being the count by which the ratio is multiplied to obtain an abundance estimate.

The intuitive rationale on which the MRR estimator was originally based implicitly assumes population and sample sizes are large enough so ratios in samples are essentially deterministic and the well-known effects of sampling variation on the bias (systematic error) of ratio estimators are negligible. When statisticians began to examine it, taking into account the probabilistic nature of sampling, the MRR estimator was found to have some highly undesirable properties (see below). Bailey [27] and Chapman [28] independently noted these problems in 1951 and proposed alternative estimators to resolve them. Chapman’s estimator is now the most commonly used and will be employed in subsequent sections of this paper.

A careful reading of Dahl’s derivation of the MRR estimator reveals that it is based on two proportions, both of which apply to the second sampling event: the proportion *r*′/*m*′ of the *m*′ marked fish present when the second sample is taken that are recaptured, and the proportion *c*′/*n*′ of the *n*′ fish present when the second sample is taken that are captured. One intuitively expects these two proportions to be approximately equal in large samples. But to arrive at the MRR estimator, we must make the additional assumption that the same individuals are present for sample 2 that were present for sample 1. Setting *r*′/*m*′ = *c*′/*n*′ and *m*′ = *m* then yields *c*′*m*/*r*′ = *n*′ = *n*, which is the basis for Eq (1). Thus, the MRR estimator (and Chapman’s modification of it) is a closed-population estimator, meaning it assumes that no addition or removal of individuals occurs between samples 1 and 2 via recruitment, death, or dispersal.

In applications to stream fisheries, the time between samples typically is one day or less [14], making it straightforward to plan assessments so the assumption of no recruitment or mortality is reasonable. The assumption of spatial closure, however, can be questionable or clearly wrong, either because block nets cannot be used to prevent dispersal or because they are not fully effective [29]. Therefore, given the widespread use of the 2-sample mark-recapture method in these applications, it is important to know what effects dispersal has on the MRR and Chapman estimators, under what conditions they are serious, and whether there are practical methods for reducing or eliminating them that do not require unique marking of individual fish (and the attendant cost of tags) or additional site visits. Some of these issues have been considered in the literature, but none adequately, as we now briefly discuss.

Ricker [30] systematically examines the effects of violating each of the other assumptions of the 2-sample mark-recapture method but has little to say about violating spatial closure. He mentions what he calls the “problem of wandering” when the study area (stream reach) does not include the entire population, but he does not quantify the effects of such dispersal on abundance estimates.

Seber [1] (p. 73) considers a situation where individuals may enter and exit the study area between samples 1 and 2. The study area contains *n* individuals when sample 1 is taken. A fraction *p* < 1 of these individuals exit between samples 1 and 2, and *I* individuals enter from outside, so that *n*′ individuals (possibly different from *n*) are in the study area when sample 2 is taken. If population and sample sizes are sufficiently large (Seber gives no guidance on how large is sufficient), the intuitive reasoning that leads to the MRR estimator, modified to account for dispersal (by assuming *m*′ = *m*(1 − *p*) and *n*′ = *n*(1 − *p*) + *I*), yields the approximation

$$\begin{array}{c} c^{\prime }m/r^{\prime }\approx n+I/\left(1-p\right)=n^{\prime }/\left(1-p\right). \end{array}$$ (2)

Thus, as long as *p* > 0 or *I* > 0 (or both), the MRR estimator overestimates one or both of the abundances *n* and *n*′ [4]. The effect of dispersal, then, seems rather simple. But these results address only a few, very simple dispersal scenarios, do not address accuracy with respect to the size of the total population of which the study-area subpopulation is a subset, and assume that population and sample sizes are large enough so bias effects of sampling variation on the MRR estimator are negligible.

The most extensive and thoughtful assessment of dispersal effects to date is by Kendall [31]. His focus is mainly on wildlife applications where the study area is a strict subset of the total population domain and one wishes to estimate total population size *n*_T_ rather than study-area abundance *n* or *n*′. Like Seber [1], he addresses situations where population and sample sizes are large enough so the effect of sampling variation on bias of the MRR estimator is negligible. But unlike Seber, he considers models where both study area and total population abundance are explicit. His main result applies to situations where dispersal thoroughly mixes the entire population between samples 1 and 2, so that all individuals in the population are equally likely to be in the study area when sample 2 is taken, regardless of where they were for sample 1. In this case, the MRR estimator applied to sampling data from the study area yields the approximation

$$\begin{array}{c} c^{\prime }m/r^{\prime }\approx n_{T}. \end{array}$$ (3)

The MRR estimator, then, accurately estimates total population size *n*_T_, which may greatly exceed study area abundances *n* and *n*′.

As this brief review illustrates, previous assessments of dispersal effects on closed-population 2-sample mark-recapture abundance estimators restrict attention to the MRR estimator (whose moments do not exist under the usual model of Bernoulli sampling [32]) and assume large population and sample sizes. Results obtained by simplifying the problem in this way are useful in stimulating intuition and suggesting qualitative patterns, but they are of limited value in applications. Indeed, that is why statistical expositions of 2-sample mark-recapture abundance estimators *without* dispersal typically employ Chapman’s estimator (whose moments exist) and permit populations and samples of any size [1, 4].

Surprisingly, then, we find that despite widespread and continuing use of closed-population 2-sample mark-recapture abundance estimators in fisheries management since circa 1900, the literature currently contains no adequate assessment of the bias of these estimators in the presence of dispersal, comparable to what is available for the case without dispersal. Results summarized above suggest that dispersal is likely to create a positive estimator bias with respect to study-area abundance with large samples from large populations, while standard results for the case without dispersal, based on Chapman’s estimator, indicate that sampling variation is likely to create a negative bias with small samples from small populations [1, 3, 4, 28, 32]. What, then, is the sign of estimator bias in small populations subject to dispersal? Under what conditions will this bias be pronounced? And how can field studies be designed to reduce its magnitude? For routine estimation of reach-scale abundance of stream fish—the main application of the 2-sample mark-recapture method today, and one where open-population methods often are not feasible—these are important questions that the literature currently does not address.

The goal of the present paper is to partially fill this knowledge gap. We provide answers to the following two questions: What are the joint effects of dispersal and sampling variability on the bias of Chapman’s estimator when population and sample sizes are not necessarily large? And are there modified sampling methods that reduce these effects without requiring unique marking of individual fish or additional site visits?

## Some background on closed populations

As background for our treatment of populations with dispersal, we briefly outline several important and well-known results for closed populations.

In stream fishery assessments, sampling for 2-sample mark-recapture studies commonly is conducted by making one or more electrofishing passes through the study reach for sample 1, and the same for sample 2 the next day. If conditions permit, block nets may be used in an attempt to isolate the reach and achieve spatial closure. The resulting number *M* of captures in sample 1 (= number of marked fish), total number *C*′ of captures in sample 2, and number *R*′ of recaptures in sample 2 are random variables.

In developing a corresponding abundance estimator, it is customary to assume that the same fish are present for both samples, the capture probability is the same for all fish during a given sampling session, and captures occur independently within and between sampling sessions. Let *n* and *n*′ denote the study-reach abundances when samples 1 and 2 are taken, let *q* and *q*′ denote the capture probabilities for samples 1 and 2, and require *n* > 0, *n*′ = *n*, 0 < *q* < 1, and 0 < *q*′ < 1. Then the number of fish captured when sampling the study reach is binomially distributed with parameters *n* and *q* for sample 1, and *n*′ = *n* and *q*′ for sample 2.

Under these assumptions, every fish falls into exactly one of four categories: captured in both samples, captured in sample 1 but not 2, captured in sample 2 but not 1, or captured in neither sample. Therefore, the probability that the two sampling sessions will produce outcome (*M*, *C*′, *R*′) = (*m*, *c*′, *r*′), subject to constitutive constraints 0 ≤ *m* ≤ *n*, 0 ≤ *r*′ ≤ *c*′ ≤ *n*, and 0 ≤ *r*′ ≤ *m*, is given by the multinomial probability mass function

$$\begin{array}{c} P\left(m,c^{\prime },r^{\prime }\right)=\frac{n!}{r^{\prime }!\left(m-r^{\prime }\right)!\left(c^{\prime }-r^{\prime }\right)!\left(n-k^{\prime }\right)!}\;\pi_{1,1}^{r^{\prime }}\;\pi_{1,0}^{m-r^{\prime }}\;\pi_{0,1}^{c^{\prime }-r^{\prime }}\;\pi_{0,0}^{n-k^{\prime }}, \end{array}$$ (4)

where *π*_1,1_ = *qq*′, *π*_1,0_ = *q*(1 − *q*′), *π*_0,1_ = (1 − *q*)*q*′, *π*_0,0_ = (1 − *q*)(1 − *q*′), and *k*′ = *m* + *c*′ − *r*′ [3]. Given the constitutive constraints just stated, this probability is positive if and only if *k*′ ≤ *n*.

If *m*, *c*′, and *r*′ are viewed as data, then Eq (4) is the likelihood function. Maximum-likelihood estimators $\hat{n}$, $\hat{q}$, and ${\hat{q}}^{\prime }$ for the parameters are

$$\begin{array}{c} \hat{n}=c^{\prime }m/r^{\prime },\;\hat{q}=r^{\prime }/c^{\prime }=m/\hat{n},\;{\hat{q}}^{\prime }=r^{\prime }/m=c^{\prime }/\hat{n} \end{array}$$

[3, 4, 28]. The MRR estimator is therefore the maximum-likelihood estimator of *n*.

Unfortunately, outcomes for which *R*′ = 0 have positive probability for any values of *C*′ and *M* such that *C*′ ≤ *n* − *M* (i.e., the number of captures in sample 2 does not exceed the number of unmarked fish in the population). For such outcomes, the denominator of the MRR estimator is 0 and the value of the estimator is undefined (infinite). It follows that the mean and higher moments of the MRR estimator do not exist, and the bias of the estimator is therefore undefined [32].

To resolve this problem, Chapman [28] proposed the alternative abundance estimator

$$\begin{array}{c} {\hat{N}}^{*}=\frac{\left(M+1\right)\left(C^{\prime }+1\right)}{R^{\prime }+1}-1. \end{array}$$

The presence of *R*′ + 1 instead of *R*′ in the denominator ensures that Chapman’s estimator is defined for all possible sampling outcomes, while the other modifications improve its bias properties [28]. (The device of adding 1 to captures and recaptures can also be used to create rigorous versions of the approximations in Eqs (2) and (3) under Bernoulli sampling, based on the strong law of large numbers.)

Chapman defined the bias of his estimator in terms of conditional mean $E({\hat{N}}^{*}|\;m,c^{\prime })$ given by

$$\begin{array}{c} E\left({\hat{N}}^{*}\;|\;m,c^{\prime }\right)=\{\begin{array}{cc} n, & \text{if}c^{\prime }⩾n-m\\ n-\frac{\left(n-m\right)!\left(n-c^{\prime }\right)!}{n!(n-m-c^{\prime }-1)!}, & \text{if}c^{\prime }<n-m \end{array} \end{array}$$ (5)

where *m* and *c*′ are the observed values of *M* and *C*′ and the expectation is taken over all possible values of *R*′. The conditional bias of ${\hat{N}}^{*}$ with respect to *n* is then defined as the difference $E({\hat{N}}^{*}|\;m,c^{\prime })-n$. It follows at once from Eq (5) that the conditional bias of Chapman’s estimator for a closed population is 0 if *c*′ ≥ *n* − *m* and is negative otherwise [3, 28, 32].

The conditional mean and bias are specific to the outcome of a particular field study and are opaque to the roles played by the capture probabilities in determining or influencing the outcome of sampling. These properties are fine if we are only interested in assessing the outcome of a single estimate from a particular field study. But they are undesirable if we are interested in planning future field studies or assessing the expected overall performance of a given sampling plan. In such cases, we wish to extend the expected value in Eq (5) to include all possible values of random variables *M* and *C*′. To this end, Skalski and Robson [3] introduce the unconditional mean and bias of Chapman’s estimator.

The key idea is to think of sampling as happening in the future instead of the past, so that only the probability distributions for the numbers of captures in samples 1 and 2 are known, not the actual values. Thus, Skalski and Robson [3] find the expected value and bias of Chapman’s estimator without conditioning on knowing the values of *M*, *C*′, or *R*′. The (unconditional) expected value $E({\hat{N}}^{*})$ of Chapman’s estimator is given by

$$\begin{array}{c} E\left({\hat{N}}^{*}\right)=\Omega_{0}-\varphi_{0}\;\lambda_{0}^{\;n-1}, \end{array}$$ (6)

where

$$\begin{array}{c} \Omega_{0}=n,\;\varphi_{0}=\left(1-q\right)\left(1-q^{\prime }\right)n,\;\lambda_{0}=1-qq^{\prime } \end{array}$$

with 0 < *φ*_0_ < *n* and 0 < λ_0_ < 1. It follows at once that $E({\hat{N}}^{*})<n$ for all *n*, but $E\left({\hat{N}}^{*}\right)\to \Omega_{0}=n$ as *n* → ∞. Ω_0_ represents the large-population, large-sample expected value of Chapman’s estimator, which equals true abundance *n*. For sufficiently high abundances in a closed study reach, then, the expected value of Chapman’s estimator is approximately the same as the value of the MRR estimator and accurately estimates study-reach abundance *n*.

The bias of estimator ${\hat{N}}^{*}$ with respect to parameter *n* is defined as $B\left({\hat{N}}^{*}\;,n\right)=E\left({\hat{N}}^{*}\right)-n$, and the relative bias with respect to *n* is defined as $b\left({\hat{N}}^{*}\;,n\right)=B\left({\hat{N}}^{*}\;,n\right)/n=\left[E\left({\hat{N}}^{*}\right)-n\right]/n$. Both measures of bias have the same sign, but relative bias is of greater interest in most applications; for example, a bias of 100 is of greater concern if true abundance is 100 (relative bias = 1) than if true abundance is 10,000 (relative bias = 0.01). From Eq (6), the relative bias is given by

$$\begin{array}{c} b\left({\hat{N}}^{*}\;,n\right)=-\left(1-q\right)\left(1-q^{\prime }\right)\;\lambda_{0}^{\;n-1}, \end{array}$$ (7)

which is always negative. Thus, Chapman’s estimator tends to underestimate abundance in closed populations. But the relative bias approaches 0 geometrically as *n* → ∞ and will be close to 0 for suitably large study-reach abundances *n*.

When abundance in the study reach is small enough so the relative bias is not negligible, its value can easily exceed 20% with realistic capture probabilities. In practice, this problem can be ameliorated by lengthening the study reach to increase *n*, increasing sampling effort to increase capture probabilities *q* and *q*′ (which decreases *φ*_0_ and λ_0_), or both. For example, with *n* = 100, the percent relative bias of ${\hat{N}}^{*}$ is about −30% if *q* = *q*′ = 0.1 and about −1% if *q* = *q*′ = 0.2. With *n* = 200, these biases are about −11% and −0.02%, respectively.

## Populations with dispersal

We now present expressions for the mean and bias of Chapman’s estimator when stochastic dispersal is permitted, with no restriction on population or sample sizes. By stochastic dispersal, we mean that the numbers of individuals entering and leaving the study reach between samples 1 and 2 are random variables rather than being fixed or deterministic quantities.

### Bias with respect to abundance in the study reach

Suppose there initially are *n* individuals in the study reach. *M* of these are caught in sample 1, marked, and released, where *M* is binomial with parameters *n* and *q*; the remaining *U* = *n* − *M* individuals remain unmarked. Between samples 1 and 2, *O*_m_ marked and *O*_u_ unmarked individuals exit the study reach and *I*_u_ unmarked individuals enter. Given *M* = *m* > 0 and *U* = *u* > 0, we assume *O*_m_ is binomial with parameters *m* and *p* and *O*_u_ is binomial with parameters *u* and *p*, where *p* ∈ (0, 1) is the exit probability (= probability that any given individual in the study reach when sample 1 is taken is not in the study reach when sample 2 is taken). To avoid unnecessarily complicating the theory, we assume that the probability of exiting the study reach between samples 1 and 2 is the same for all fish in the study reach, and we interpret this shared exit probability as the weighted average of spatially-explicit exit probabilities within the reach. *I*_u_ is a random variable as well, but for present purposes we need assume only that it has finite mean $\tilde{I}⩾0$. When sample 2 is taken, then, there are *N*′ = *n* − *O*_m_ − *O*_u_ + *I*_u_ individuals in the study reach, of which *M*′ = *M* − *O*_m_ are marked and *U*′ = *U* − *O*_u_ + *I*_u_ = *N*′ − *M*′ are unmarked.

Under these assumptions, the expected value of abundance *N*′ when sample 2 is taken is $E\left(N^{\prime }\right)=n\left(1-p\right)+\tilde{I}$, and the expected value of ${\hat{N}}^{*}$ is given by

$$\begin{array}{c} E\left({\hat{N}}^{*}\right)=\Omega_{1}-\varphi_{1}\;\lambda_{1}^{\;n-1}, \end{array}$$ (8)

where

$$\begin{array}{ccc} \Omega_{1} & = & n+\tilde{I}/\left(1-p\right)=E\left(N^{\prime }\right)/\left(1-p\right)\\ \varphi_{1} & = & \left(\Omega_{1}[1-qq^{\prime }\left(1-p\right)]-nq[1-q^{\prime }\left(1-p\right)])[1-q^{\prime }\left(1-p\right)\right]\\ \lambda_{1} & = & 1-qq^{\prime }\left(1-p\right) \end{array}$$

with 0 < λ_1_ < 1 and 0 < *φ*_1_ < Ω_1_ (S1 File).

We note that while there is no logically necessary relationship between $\tilde{I}$, *p*, and *n*, these quantities are connected in real streams by the physical and behavioral mechanisms by which dispersal occurs. For example, lengthening the study reach will increase *n* but also will decrease *p*, because the average distance that must be traveled to exit the study-reach increases. And increasing overall abundance in the stream segment in which the study reach is embedded will increase *n* but also will increase $\tilde{I}$, because the density of fish (number per unit area) in the source areas for immigrants increases. We return to this point in the numerical examples below.

Notice that all terms in Eq (8) reduce to those in Eq (6) if we set $p=0=\tilde{I}$. It is also apparent in Eq (8) that $E\left({\hat{N}}^{*}\right)\to \Omega_{1}=n+\tilde{I}/\left(1-p\right)$ as *n* → ∞. This result shows that as study-reach abundance *n* becomes large, the expected value of Chapman’s estimator converges to Seber’s large-population, large-sample expression for the MRR estimator with dispersal stated in Eq (2). Similar to the case of a closed study reach, then, the expected value of Chapman’s estimator will be approximately the same as the value of the MRR estimator if the initial study-reach abundance (when sample 1 is taken) is sufficiently high.

The relative bias of Chapman’s estimator with respect to initial study-reach abundance *n* is given by

$$\begin{array}{c} b\left({\hat{N}}^{*}\;,n\right)=\tilde{I}/\left[(1-p)n\right]-\left(\varphi_{1}/n\right)\lambda_{1}^{n-1}, \end{array}$$

which has two terms. The first term reflects only the bias effect of dispersal; it coincides with the relative bias with respect to *n* of the MRR estimator with dispersal (= *I*/[(1 − *p*)*n*], from Eq (2)) and has no analog in the relative bias of Chapman’s estimator for a closed population given by Eq (7). The second term mainly reflects the bias effect of sampling variation in a finite population; it has no analog in the relative bias of the MRR estimator with dispersal but does have an analog in the relative bias of Chapman’s estimator for a closed population. Depending on the relative sizes of these two terms, $b({\hat{N}}^{*}\;,n)$ can be positive, negative, or zero (see numerical examples below).

With stochastic dispersal, study-reach abundance *N*′ when sample 2 is taken is a random variable with expected value E(*N*′). Though E(*N*′) is not a parameter, it is still necessary to consider whether Chapman’s estimator tends to systematically over- or underestimate the abundance when sample 2 is taken. Using the expected value of this abundance as the benchmark for assessing accuracy, it is convenient to refer to the difference between $E({\hat{N}}^{*})$ and E(*N*′) as the bias of Chapman’s estimator with respect to E(*N*′). With this terminology, the relative bias of ${\hat{N}}^{*}$ with respect the E(*N*′) is given by

$$\begin{array}{c} b\left({\hat{N}}^{*}\;,E\left(N^{\prime }\right)\right)=p/\left(1-p\right)-[\varphi_{1}/E\left(N^{\prime }\right)]\lambda_{1}^{n-1}. \end{array}$$

Like $b({\hat{N}}^{*}\;,n)$, $b({\hat{N}}^{*}\;,E\left(N^{\prime }\right))$ has two terms, the first representing the bias effect of dispersal and coinciding with the relative bias with respect to *n*′ of the MRR estimator (= *p*/(1 − *p*), from Eq (2)) and the second mainly reflecting the bias effect of sampling variation and having an analog in the relative bias of Chapman’s estimator for a closed population. As in the case of relative bias with respect to study-reach abundance *n* when sample 1 is taken, the relative bias with respect to the expected value E(*N*′) of study-reach abundance when sample 2 is taken can be postive, negative, or zero, depending on the relative sizes of these two terms (see numerical examples below).

### Bias with respect to total population size

In applications to stream fisheries, the study reach typically includes only a subset of the total population. Suppose, then, that the total population also includes fish in a reach extending upstream of the study reach and in another reach extending downstream of the study reach, and that the total population is closed between samples 1 and 2. Denote the three reaches that constitute the total population domain by S (study reach), US (upstream of S), and DS (downstream of S), and let the corresponding numbers of fish when sample 1 is taken be *n*, *n*_US_, and *n*_DS_. Then the total population size nT when sample 1 is taken is given by *n*_T_ = *n* + *n*_US_ + *n*_DS_.

To address bias of Chapman’s estimator with respect to total population size *n*_T_ using the above theory, suppose zones US and DS are small enough so it is reasonable to assume that every fish in zone US when sample 1 is taken has probability *p*_US,S_ < 1 of being in the study reach for sample 2, and every fish in zone DS when sample 1 is taken has probability *p*_DS,S_ < 1 of being in the study reach for sample 2. Let *p*_S,S_ = 1 − p denote the probability that any given fish in the study reach when sample 1 is taken will be there again when sample 2 is taken. In Eq (8), then, $\tilde{I}=n_{\text{US}}p_{\text{US},S}+n_{\text{DS}}p_{\text{DS},S}$ and terms Ω_1_, *φ*_1_, and λ_1_ have forms

$$\begin{array}{ccc} \Omega_{1} & = & n+(n_{\text{US}}p_{\text{US},S}+n_{\text{DS}}p_{\text{DS},S})/p_{S,S}=E\left(N^{\prime }\right)/p_{S,S}\\ \varphi_{1} & = & \left[\Omega_{1}(1-qq^{\prime }p_{S,S})-nq(1-q^{\prime }p_{S,S})](1-q^{\prime }p_{S,S}\right)\\ \lambda_{1} & = & 1-qq^{\prime }p_{S,S}. \end{array}$$

Using these expressions in Eq (8) and defining the bias of ${\hat{N}}^{*}$ with respect to parameters *n* and *n*_T_ in the usual way, the following results are evident:

1. If *p*_S,S_ > max {*p*_US,S_, *p*_DS,S_}, so that fish that were in the study reach when sample 1 was taken are more likely to be there when sample 2 is taken than are fish that were outside the study reach when sample 1 is taken, then ${\hat{N}}^{*}$ is negatively biased with respect to *n*_T_.
2. If *p*_S,S_ < min {*p*_US,S_, *p*_DS,S_}, so that fish that were in the study reach when sample 1 was taken are *less* likely to be there when sample 2 is taken than are fish that were outside the study reach when sample 1 is taken, then ${\hat{N}}^{*}$ can be positively or negatively biased (or unbiased) with respect to *n*_T_ but will be positively biased for sufficiently large *n*.
3. If *p*_*j*,S_ = *p*_S_ for all *j* ∈ {US, S, DS}, so that all fish are equally likely to be in the study area when sample 2 is taken, regardless of their location when sample 1 was taken, then ${\hat{N}}^{*}$ is negatively biased with respect to *n*_T_, but the bias becomes negligible for sufficiently large *n*.

Property 1 is the most relevant for field applications, with the other properties mainly illustrating the range of theoretical possibilities. Property 3 addresses the extreme case where every individual in the population has the same chance of being in the study reach when sample 2 is taken. Dispersal is then so extensive that it thoroughly mixes the entire population between samples (Kendall [31] calls this completely random dispersal). In this case, Chapman’s estimator is negatively biased with respect to total population size *n*_T_. The bias is negligible, however, if study-reach abundance *n* (hence *n*_T_) is sufficiently large, consistent with Kendall’s large-population result stated in Eq (3).

All the above properties rely on the assumption that the total population domain is not much larger than the study reach. When the population domain greatly exceeds the study reach, it is unreasonable to assume that all individuals upstream (or downstream) of the study reach have the same probability of entering the study reach between samples. In this case, however, we may interpret zones US and DS as containing all the fish likely to enter the study reach between samples 1 and 2, and to restrict the equal-probability assumption to these fish. The above properties still apply to cumulative abundance *n* + *n*_US_ + *n*_DS_ in zones US, S, and DS, but this will be less than the total population size.

## A modified sampling scheme that reduces dispersal-related bias

A common method for dealing with dispersal in streams when block nets cannot be used or are not fully effective is simply to lengthen the study reach, with sampling being conducted throughout the expanded reach in the usual way [33]. The intent is to decrease the proportion of marked fish that exit the study reach between samples 1 and 2 and to decrease the proportion of fish present when sample 2 is taken that entered from outside the reach following sample 1. This approach increases the area that must be sampled on both sampling occasions, but it avoids the need to uniquely mark individual fish and still requires only two days to complete.

**Fig 1:**
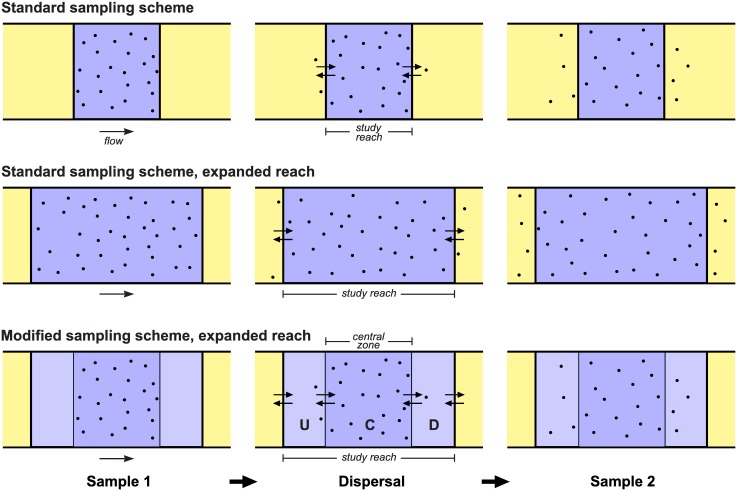
Comparison of standard and modified sampling schemes applied to a stream reach subject to dispersal. The study reach is shown in tints of blue, parts of the population domain outside the study reach in yellow. Black dots: fish marked and released in sample 1 (unmarked fish not shown). Top row: Standard scheme applied to a short study reach. Middle row: Standard scheme applied to an expanded study reach. Bottom row: Modified scheme applied to an expanded study reach, with zones U, C, and D indicated (darker blue: samples 1 and 2 are taken; lighter blue: only sample 2 is taken). Left panels show locations of marked fish upon completion of sample 1. Center panels show dispersal of marked fish in progress. Right panels show locations of marked fish when sample 2 is taken.

As an alternative, we propose the following modified sampling scheme. The original study reach is lengthened by moving its upstream boundary further upstream and its downstream boundary further downstream (Fig 1). The expanded study reach now comprises three zones: a central zone (the original study reach), a new upstream zone, and a new downstream zone. Call these zones U (upstream), C (central), and D (downstream). Sample 1 is taken only from zone C, and all captured fish are returned there after marking; sample 2 is taken from the entire study reach. If zones U and D are long enough and the time between samples 1 and 2 is short enough relative to typical movement rates of the fish species being studied, then none or very few of the marked fish (all initially located in zone C) will leave the expanded study reach before sample 2 is taken. Therefore *M*′ ≈ *M* and the effect of dispersal on bias of Chapman’s estimator should be substantially reduced.

It is simple to confirm this idea for the large-population, large-sample case by modifying Dahl’s argument presented in the Introduction. Recall that his argument is based on the intuitive notion that, with a large sample from a large population, we should have *r*′/*m*′ ≈ *c*′/*n*′, where *r*′ is the number of marked fish captured in sample 2, *m*′ is the number of marked fish still present in the study reach when sample 2 is taken, *c*′ is the total number of captures in sample 2, and *n*′ is abundance in the study reach when sample 2 is taken. With dispersal, *n*′ ≈ *n*(1 − *p*) + *I*, as in Seber’s argument that leads to Eq (2). But with the modified sampling scheme, we have *m*′ ≈ *m* instead of *m*′ = *m*(1 − *p*). It follows that, instead of Eq (2), the large-population, large-sample approximation for the MRR is

$$\begin{array}{c} c^{\prime }m/r^{\prime }\approx n^{\prime }=n\left(1-p\right)+I. \end{array}$$ (9)

With the modified sampling scheme, then, the MRR accurately estimates abundance *n*′ in the study reach when sample 2 is taken, even if substantial dispersal occurs.

We now consider this intuitive idea in the context of populations and samples that are not necessarily large, where the bias effect of sampling variation must be accounted for.

### Bias with respect to abundance in the study reach

With the modified sampling scheme, sample 1 is taken only from central zone C, while sample 2 is taken from the entire study reach comprising zones U, C, and D (upstream to downstream order). Let *n*_U_, *n*_C_, and *n*_D_ be the numbers of fish in the three zones of the study reach when sample 1 is taken, and let *n* = *n*_U_ + *n*_C_ + *n*_D_ be the total number of fish in the study reach. Assume that each fish in zone *j* ∈ {U, C, D} when sample 1 is taken has probability *p*_*j*_ of exiting the entire study reach before sample 2 is taken, and let $\tilde{I}$ denote the expected value of the number of (unmarked) fish entering the study reach from outside between samples 1 and 2. Then the expected value of the number *N*′ of fish in the study reach when sample 2 is taken is given by

$$\begin{array}{c} E\left(N^{\prime }\right)\;=\;n-n\left(\pi_{U}p_{U}+\pi_{C}p_{C}+\pi_{D}p_{D}\right)+\tilde{I}\;=\;n\left(1-\tilde{p}\right)+\tilde{I}, \end{array}$$

where *π*_*j*_ = *n*_*j*_/*n* and $\tilde{p}=\pi_{U}p_{U}+\pi_{C}p_{C}+\pi_{D}p_{D}$. It is straightforward to show that the expected value of Chapman’s estimator is given by

$$\begin{array}{c} E\left({\hat{N}}^{*}\right)=\Omega_{2}-\varphi_{2}\;\lambda_{2}^{\;n_{C}-1}, \end{array}$$ (10)

where

$$\begin{array}{lll} \Omega_{2} & = & \left[n\left(1-\tilde{p}\right)+\tilde{I}\right]/\left(1-p_{\text{C}}\right)=\text{E}\left(N^{\prime }\right)/\left(1-p_{\text{C}}\right)\\ \varphi_{2} & = & \left(\Omega_{2}\left[1-qq^{\prime }\left(1-p_{\text{C}}\right)]-n_{\text{C}}q[1-q^{\prime }\left(1-p_{\text{C}}\right)\right]\right)\left[1-q^{\prime }\left(1-p_{\text{C}}\right)\right]\\ \lambda_{2} & = & 1-qq^{\prime }\left(1-p_{\text{C}}\right) \end{array}$$

with 0 < *φ*_2_ < Ω_2_ and 0 < λ_2_ < 1 (S1 File). Note that all terms in Eq (10) reduce to those in Eq (8) if we remove zones U and D by setting *n*_*U*_ = 0 = *n*_*D*_, *n*_*C*_ = *n*, and *p*_*C*_ = *p*. We also note that, as with the standard sampling scheme, $\tilde{I}$ and $\tilde{p}$ are not necessarily independent of *n* (see the numerical examples below).

When the modified sampling scheme is implemented properly, *p*_C_ ≈ 0. Setting *p*_C_ = 0, the above formulas for Ω_2_, *φ*_2_, and λ_2_ simplify to

$$\begin{array}{ccc} \Omega_{2} & = & n\left(1-\tilde{p}\right)+\tilde{I}=E\left(N^{\prime }\right)\\ \varphi_{2} & = & [\Omega_{2}\left(1-qq^{\prime }\right)-n_{C}q\left(1-q^{\prime }\right)]\left(1-q^{\prime }\right)\\ \lambda_{2} & = & 1-qq^{\prime }. \end{array}$$

As *n*_C_ → ∞, $E\left({\hat{N}}^{*}\right)∼\Omega_{2}=E\left(N^{\prime }\right)=n\left(1-\tilde{p}\right)+\tilde{I}$. The expected value of Chapman’s estimator therefore converges to the value of the large-population, large-sample expression for the MRR estimator with dispersal and the modified sampling scheme stated in Eq (9).

Defining the relative bias of ${\hat{N}}^{*}$ with respect to *n* and E(*N*′) as above, we find at once from Eq (10) with *p*_C_ = 0 that the relative bias with respect to *n* is given by

$$\begin{array}{c} b\left({\hat{N}}^{*}\;,n\right)=-p+\tilde{I}/n-\left(\varphi_{2}/n\right)\lambda_{2}^{\;n_{C}-1}, \end{array}$$

and the relative bias with respect to E(*N*′) is given by

$$\begin{array}{c} b\left({\hat{N}}^{*}\;,E\left(N^{\prime }\right)\right)=-[\varphi_{2}/E\left(N^{\prime }\right)]\lambda_{2}^{\;n_{C}-1}. \end{array}$$

As in the case of the standard sampling scheme, the relative bias $b({\hat{N}}^{*}\;,n)$ with respect to initial study-area abundance has two terms. The first represents the bias effect of unbalanced dispersal; it is positive, negative, or zero according as the expected value $\tilde{I}$ of the number of immigrants between samples 1 and 2 is greater than, less than, or equal to the expected value *np* of the number of emigrants. The second term reflects sampling variation and approaches 0 as zone-C abundance *n*_C_ becomes large. If dispersal is unbalanced, then, the asympotic relative bias with respect to initial study-reach abundance *n* will be either positive or negative, not zero. By contrast, the relative bias $b({\hat{N}}^{*}\;,E\left(N^{\prime }\right))$ with respect to the expected value of study-reach abundance when sample 2 is taken has only a term reflecting the bias effect of sampling variation, which again approaches 0 as *n*_C_ becomes large. The asymptotic relative bias with respect to E(*N*′) is therefore zero. Broadly speaking, then, ${\hat{N}}^{*}$ provides a more-accurate estimate of the expected value of study-reach abundance *N*′ when sample 2 is taken than of study reach abundance *n* when sample 1 was taken unless dispersal is balanced, especially when *n*_C_ and *n* are large. If *n*_C_ and *n* are large and the modified sampling scheme is properly implemented, then $E\left({\hat{N}}^{*}\right)\approx E\left(N^{\prime }\right)$, regardless of whether dispersal is balanced.

### Bias with respect to total population size

We address bias with respect to total population size in the same way as for the standard sampling scheme, retaining the assumption that the total population is closed between samples 1 and 2. The study reach S now comprises zones U, C, and D, while the total population domain comprises the study reach, a reach US upstream of S, and a reach DS downstream of S. When sample 1 is taken, the total population size is *n*_T_ = *n* + *n*_US_ + *n*_DS_, where *n* = *n*_U_ + *n*_C_ + *n*_D_. We assume that every fish in reach or zone *j* ∈ {US, U, C, D, DS} when sample 1 is taken has probability *p*_*j*,S_ of being in the study reach when sample 2 is taken, with *p*_US,S_, *p*_DS,S_ < 1. Then in Eq (10), $\tilde{I}=n_{\text{US}}p_{\text{US},S}+n_{\text{DS}}p_{\text{DS},S}$ and terms Ω_2_, *φ*_2_, and λ_2_ have forms

$$\begin{array}{ccc} \Omega_{2} & = & (n{\tilde{p}}_{S,S}+n_{\text{US}}p_{\text{US},S}+n_{\text{DS}}p_{\text{DS},S})/p_{C,S}=E\left(N^{\prime }\right)/p_{C,S}\\ \varphi_{2} & = & \left[\Omega_{2}(1-qq^{\prime }p_{C,S})-n_{C}q(1-q^{\prime }p_{C,S})](1-q^{\prime }p_{C,S}\right)\\ \lambda_{2} & = & 1-qq^{\prime }p_{C,S} \end{array}$$

with ${\tilde{p}}_{S,S}=\pi_{U}p_{U,S}+\pi_{C}p_{C,S}+\pi_{D}p_{D,S}$. The following properties of the bias of ${\hat{N}}^{*}$ with respect to *n*_T_ are evident from Eq (10):

1. If *p*_C,S_ > max{*p*_*j*,S_, *j* ≠ C}, so that fish that were in zone C when sample 1 was taken are more likely than other fish to be in the study reach when sample 2 is taken, then 0 < Ω_2_ < *n*_T_. In this case, ${\hat{N}}^{*}$ is negatively biased with respect to *n*_T_ for all *n*_C_ and remains so even in the limit as *n*_C_, *n* → ∞.
2. In the special case where the modified sampling scheme is improperly implemented and *p*_*j*,S_ = *p*_S_ for all *j*, so that all fish are equally likely to be in the study reach when sample 2 is taken, we have Ω_2_ = *n*_T_. In this case, ${\hat{N}}^{*}$ is negatively biased with respect to *n*_T_ for all *n*_C_, but the bias becomes negligible for sufficiently large *n*_C_ (and hence *n*, *n*_T_).

Property 1 implies that if the modified sampling scheme is properly implemented, Chapman’s estimator will be negatively biased with respect to total population size *n*_T_, regardless of the number of fish in the central zone when sample 1 is taken. Property 2 is of little relevance to field applications but provides a link to Kendall’s result in Eq (3).

## Comparison of the standard and modified sampling schemes

In this section, we present numerical results that permit quantitative comparison of the relative biases of Chapman’s estimator for three sampling implementations: the standard sampling scheme applied to the original study reach, the standard scheme applied to the expanded study reach, and the modified scheme applied to the expanded study reach. All comparisons are based on a range of different abundances, capture probabilities, and dispersal rates using Eqs (8) and (10). To make the comparisons fair and informative, it is necessary to standardize sampling effort so we are assessing different choices of study-reach length and sampling scheme instead of different levels of sampling effort, and to represent dispersal in such a way that both the probability of exiting the study reach and the expected value of the number of fish entering the study reach from outside between samples 1 and 2 differ between study-reach lengths and sampling schemes in an appropriate way that does not favor one sampling implementation over another. Before presenting the numerical results, we briefly explain how these requirements were satisfied.

**Fig 2:**
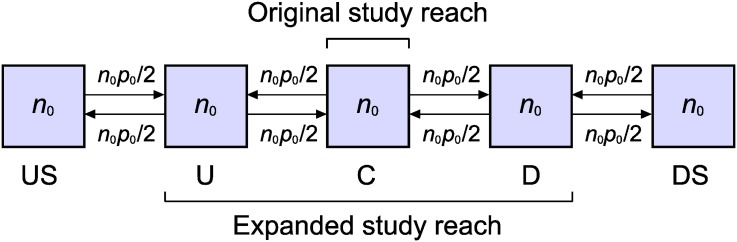
Definition sketch for comparing bias of standard and modified sampling schemes. Five equal-size zones of a stream reach are shown. Zone C is the original study reach; zones U, C, and D constitute the expanded study reach. Each zone contains *n*_0_ fish when sample 1 is taken. Isotropic dispersal occurs between samples 1 and 2, with probability *p*_0_ of exiting any given zone. See text for additional details.

Each sampling implementation can be viewed as occurring in one of three identical stream segments, each of which comprises five zones. We focus on the case where all zones have the same length and the same initial abundance *n*_0_ when sample 1 is taken (Fig 2). To ensure that the exit probabilities and mean numbers of immigrants in Eqs (8) and (10) vary appropriately among study-reach lengths and zones, we employ a simple dispersal model in which fish abundance and density are spatially uniform and dispersal is isotropic and local. More specifically, the probability that any given fish in any particular zone exits that zone between samples 1 and 2 is *p*_0_, with probability *p*_0_/2 of exiting in the upstream direction and probability *p*_0_/2 of exiting in the downstream direction. Fish can disperse only to an adjacent zone. The expected value of the number of fish dispersing from any particular zone to the adjacent upstream (or downstream) zone between samples 1 and 2 is therefore *n*_0_ *p*_0_/2, and the expected value of the change in number of fish within any particular study-reach zone is therefore 0.

Of the five zones in each stream segment, the central one is the study reach when the original study reach is employed, but it is zone C when the expanded study reach is employed. With the original study reach, then, study-reach abundance when sample 1 is taken is *n* = *n*_0_, and the zones labeled U and D in Fig 2 are the sources of immigrants between samples 1 and 2. The expanded study reach comprises zones U, C, and D; the study-reach abundance when sample 1 is taken is *n* = 3*n*_0_, and zones US and DS are the sources of immigrants between samples.

The probability *p* of a randomly chosen fish exiting the original study reach is the probability of exiting a single zone, so *p* = *p*_0_ in Eq (8). The probability of a randomly chosen fish exiting the expanded study reach with either the standard or modified sampling scheme is the weighted average $\tilde{p}$ of the probabilities of exiting the study reach from zones U, C, and D. Since the probability of exiting the study reach from zone C is 0, $\tilde{p}=\pi_{U}p_{0}/2+\pi_{D}p_{0}/2=p_{0}/3$ in Eq (10). The expected value of the total number of fish exiting the study reach between samples 1 and 2 is *np* = *n*_0_ *p*_0_ for the original study reach and $n\tilde{p}=n_{0}p_{0}$ for the expanded reach. The expected value of the number of fish entering the study reach is $\tilde{I}=n_{0}p_{0}$ (*n*_0_ *p*_0_/2 from each end) for both sampling schemes. Therefore, E(*N*′) = *n* (balanced dispersal), regardless of the sampling implementation.

We assume that samples are taken actively (e.g., by electrofishing). To ensure that sampling effort (as measured by duration of sampling) is the same when sampling is restricted to the original study reach or zone C as when all three zones are sampled, it is necessary to adjust the capture probability for zone C as follows. First, note that with constant search rate and capture efficiency, capture probability *q* (or *q*′) can be expressed as a function of sampling duration *T* as

$$\begin{array}{c} q\left(T\right)=1-e^{-\alpha rT/V} \end{array}$$

(e.g., [34]), where *α* is the capture efficiency (proportion of encountered fish caught), *r* is the search rate (volume or area searched per unit time), and *V* is the size (volume or area) of the zone or study reach being sampled. If the size *V*_X_ of the expanded study reach is *k* times the size *V*_C_ of zone C, then *V*_C_ = *V*_X_/*k* and the corresponding capture probabilities *q*_C_ and *q*_X_ with fixed sampling duration *T* are related as follows:

$$\begin{array}{c} q_{C}=1-e^{-\alpha rT/V_{C}}=1-e^{-k\alpha rT/V_{X}}=1-{\left(1-q_{X}\right)}^{k}. \end{array}$$ (11)

Thus, with constant sampling effort (e.g., 3 hours of electrofishing), one sampling pass through the expanded study reach is equivalent to *k* passes through zone C (e.g., 1 hour per pass). This relationship holds for both samples 1 and 2 when comparing the standard sampling scheme in the original and expanded study reaches, and for sample 1 when comparing the modified and standard schemes in the expanded study reach.

Table 1 compares the percent relative biases $100\times b\left({\hat{N}}^{*}\;,n\right)=100\times b\left({\hat{N}}^{*}\;,E\left(N^{\prime }\right)\right)$ of Chapman’s estimator for the standard sampling scheme applied to an original study reach of length 30 (arbitrary units), the standard scheme applied to an expanded study reach of length 90 (so *k* = 3), and the modified scheme applied to an expanded study reach of length 90. Capture probabilities were deliberately chosen to span a wide range and vary as follows: five “baseline” capture probabilities *q*_0_ are examined for all cases: *q*_0_ = 0.1, 0.3, 0.5, 0.7, 0.9. For the expanded study reach, the capture probabilities for samples 1 and 2 with the standard sampling scheme are the same as the baseline capture probabilities, so *q* = *q*′ = *q*_0_. The same area is sampled for sample 2 with the modified sampling scheme, so *q*′ = *q*_0_ for this sample, too. For both samples with the standard sampling scheme in the original study reach, and for sample 1 with the modified scheme, the area sampled is only one-third as large. Therefore, holding sampling effort constant, the capture probabilities for samples 1 and 2 in the original study reach are *q* = *q*′ = 1 − (1 − *q*_0_)^3^, and the capture probability for sample 1 with the modified scheme is *q* = 1 − (1 − *q*_0_)^3^. The probability of exiting a single zone between samples is *p*_0_ = 0.1, 0.3, 0.5. Abundance in each of zones US, U, C, D, DS is *n*_0_ = 50, 100, 200, 300, so study-reach abundance is *n* = *n*_0_ and *n* = 3*n*_0_ with the original and expanded study reaches, respectively.

**Table 1:** Comparison of standard and modified sampling schemes.

*n*_0_	*q*_0_	Standard scheme, original reach					Standard scheme, expanded reach					Modified scheme, expanded reach				
		*q*	*q*′	PRB for various *p*_0_			*q*	*q*′	PRB for various *p*_0_			*q*	*q*′	PRB for various *p*_0_		
				0.1	0.3	0.5			0.1	0.3	0.5			0.1	0.3	0.5
50	0.100	0.271	0.271	8.9	35.9	76.6	0.100	0.100	-16.4	-12.8	-8.9	0.271	0.100	-20.9	-20.9	-20.9
	0.300	0.657	0.657	11.1	42.9	100.0	0.300	0.300	3.5	11.1	20.0	0.657	0.300	-0.0	-0.0	-0.0
	0.500	0.875	0.875	11.1	42.9	100.0	0.500	0.500	3.5	11.1	20.0	0.875	0.500	-0.0	-0.0	-0.0
	0.700	0.973	0.973	11.1	42.9	100.0	0.700	0.700	3.5	11.1	20.0	0.973	0.700	0.0	0.0	0.0
	0.900	0.999	0.999	11.1	42.9	100.0	0.900	0.900	3.5	11.1	20.0	0.999	0.900	0.0	0.0	0.0
100	0.100	0.271	0.271	11.0	42.4	96.4	0.100	0.100	-1.2	5.0	11.8	0.271	0.100	-5.3	-5.3	-5.3
	0.300	0.657	0.657	11.1	42.9	100.0	0.300	0.300	3.5	11.1	20.0	0.657	0.300	-0.0	-0.0	-0.0
	0.500	0.875	0.875	11.1	42.9	100.0	0.500	0.500	3.5	11.1	20.0	0.875	0.500	0.0	0.0	0.0
	0.700	0.973	0.973	11.1	42.9	100.0	0.700	0.700	3.5	11.1	20.0	0.973	0.700	0.0	0.0	0.0
	0.900	0.999	0.999	11.1	42.9	100.0	0.900	0.900	3.5	11.1	20.0	0.999	0.900	0.0	0.0	0.0
200	0.100	0.271	0.271	11.1	42.9	99.9	0.100	0.100	3.2	10.7	19.3	0.271	0.100	-0.3	-0.3	-0.3
	0.300	0.657	0.657	11.1	42.9	100.0	0.300	0.300	3.5	11.1	20.0	0.657	0.300	0.0	0.0	0.0
	0.500	0.875	0.875	11.1	42.9	100.0	0.500	0.500	3.5	11.1	20.0	0.875	0.500	0.0	0.0	0.0
	0.700	0.973	0.973	11.1	42.9	100.0	0.700	0.700	3.5	11.1	20.0	0.973	0.700	0.0	0.0	0.0
	0.900	0.999	0.999	11.1	42.9	100.0	0.900	0.900	3.5	11.1	20.0	0.999	0.900	0.0	0.0	0.0
300	0.100	0.271	0.271	11.1	42.9	100.0	0.100	0.100	3.4	11.1	19.9	0.271	0.100	-0.0	-0.0	-0.0
	0.300	0.657	0.657	11.1	42.9	100.0	0.300	0.300	3.5	11.1	20.0	0.657	0.300	0.0	0.0	0.0
	0.500	0.875	0.875	11.1	42.9	100.0	0.500	0.500	3.5	11.1	20.0	0.875	0.500	0.0	0.0	0.0
	0.700	0.973	0.973	11.1	42.9	100.0	0.700	0.700	3.5	11.1	20.0	0.973	0.700	0.0	0.0	0.0
	0.900	0.999	0.999	11.1	42.9	100.0	0.900	0.900	3.5	11.1	20.0	0.999	0.900	0.0	0.0	0.0

Comparison of percent relative bias (PRB) for the standard sampling scheme applied to an original study reach of length 30 (arbitrary units) containing 50, 100, 200, or 300 fish, and the standard and modified sampling schemes applied to an expanded study reach of length 90 containing 150, 300, 600, or 900 fish. *n*_0_ is abundance in the original study reach and each zone of the expanded reach when sample 1 is taken. *q*_0_ is the capture probability for samples 1 and 2 for the standard sampling scheme with the expanded study reach; for other cases, capture probabilities *q* for sample 1 and *q*′ for sample 2 were adjusted as necessary to make sampling effort equal for all cases. PRB values are shown for three values of the probability *p*_0_ of exiting zone US, U, C, D, or DS between samples. See text for further explanation.

Note that with the highest single-zone exit probability (*p*_0_ = 0.5, implying $\tilde{p}\approx 0.17$), the relative bias of Chapman’s estimator is roughly 100% in most cases where the standard scheme is applied to the original study reach. Tripling the size of the study reach while maintaining the same level of sampling effort reduces the relative bias to roughly 20% in most cases when the standard scheme is retained but essentially eliminates it in most cases when the modified scheme is used. The case with the lowest capture probability (*q*_0_ = 0.1) and zone-C abundance (*n*_0_ = 50) is the only one where the standard sampling scheme performs better than the modified scheme. The reason for the poor performance of the modified scheme in this case is that the combination of low capture probability and low zone-C abundance when sample 1 is taken magnifies sampling variability, which, as noted in the Introduction, is known to produce negative bias in 2-sample mark-recapture estimators.

Bias differences between the three sampling implementations are less pronounced at lower values of zone-exit probability *p*_0_. In most cases, however, the standard sampling scheme applied to the expanded study reach performs better than the standard scheme applied to the original study reach, and the modified scheme applied to the expanded study reach performs even better. Broadly speaking, the numerical results in Table 1 show that the higher the dispersal probability is, the greater is the advantage of expanding the study reach and using the modified sampling scheme. The few exceptions occur in cases where the single-zone abundance is 100 or less and, simultaneously, the sample-2 capture probability is 0.1 in the expanded reach. These results indicate that the advantage of expanding the study reach with fixed sampling effort disappears if the capture probability is roughly 0.1 (or lower, presumably) and the single-zone abundance is roughly 100 or lower.

## How large is large?

Eqs (8) and (10) can be rearranged to yield expressions for the bias of Chapman’s estimator that are valid for any population or sample size. Because λ_*i*_ ∈ (0, 1), the geometric term in each of these equations approaches 0 as *n* → ∞ or *n*_C_ → ∞, and the biases consequently converge to asymptotic forms that are valid only for large populations and samples. The question we now ask is: How large must abundance *n* be to ensure that these asymptotic forms provide good approximations to the exact values based on Eqs (8) and (10)?

A thorough investigation of this problem is beyond the scope of the present paper, but we can obtain reasonable numerical estimates using the model of abundance and dispersal presented in the previous section. With this model, the expected value of the number of fish exiting any zone of the stream between samples 1 and 2 is the same as the expected value of the number entering, implying that E(*N*′) = *n*. Therefore, the bias of Chapman’s estimator with respect study-reach abundance *n* when sample 1 is taken is the same as the bias with respect to the expected value of study-reach abundance E(*N*′) when sample 2 is taken, and we need not distinguish between these two ways of expressing bias.

In the context of this model, Eqs (8) and (10) imply that relative bias $b\left({\hat{N}}^{*}\;,n\right)=b\left({\hat{N}}^{*}\;,E\left(N^{\prime }\right)\right)$ for both the standard and modified sampling schemes has the form

$$\begin{array}{c} b\left({\hat{N}}^{*}\;,n\right)=b_{\infty }-\beta \lambda^{n_{x}-1} \end{array}$$

where *n*_*x*_ = *n* for the standard sampling scheme and *n*_*x*_ = *n*_C_ for the modified scheme, *b*_∞_ is the asymptotic relative bias as *n*_*x*_ → ∞, and λ is λ_1_ for the standard scheme and λ_2_ for the modified scheme. For the standard scheme, we find from Eq (8) that *b*_∞_ = *p*/(1 − *p*) and

$$\begin{array}{c} \beta =(\frac{1-qq^{\prime }\left(1-p\right)}{1-p}-q\left[1-q^{\prime }\left(1-p\right)\right])\left[1-q^{\prime }\left(1-p\right)\right]. \end{array}$$

For the modified scheme, we find that *b*_∞_ = 0 and

$$\begin{array}{c} \beta =\left[1-qq^{\prime }-q\left(1-q^{\prime }\right)/3\right]\left(1-q^{\prime }\right). \end{array}$$

As noted in the previous section, the exit probability for the original study reach is *p* = *p*_0_ and the exit probability for the expanded study reach is $p=\tilde{p}=p_{0}/3$. Also as in the previous section, baseline capture probability *q*_0_ is the capture probability for both samples with the standard scheme in the expanded study reach and for sample 2 with the modified scheme. Holding sampling effort constant, the capture probability for both samples with the standard scheme in the original study reach and for sample 1 with the modified scheme is 1 − (1 − *q*_0_)^3^.

Let us agree to say that asymptotic relative bias *b*_∞_ adequately approximates exact value *b* if |*b* − *b*_∞_| ≤ 10^−2^. We ask: How large must study-reach abundance *n* be to ensure that this inequality is satisfied? For the standard sampling scheme, we find that we must require

$$\begin{array}{c} n\geq n_{\text{crit}}=1+\frac{2+{\text{log}}_{10}\left(\beta \right)}{|{\text{log}}_{10}\left(\lambda_{1}\right)|}, \end{array}$$

while for the modified scheme, with *n* = 3*n*_C_, we must require

$$\begin{array}{c} n\geq n_{\text{crit}}=3(1+\frac{2+{\text{log}}_{10}\left(\beta \right)}{|{\text{log}}_{10}\left(\lambda_{2}\right)|}). \end{array}$$

Table 2 shows values of *n*_crit_ and asymptotic percent relative bias *PRB*_∞_ = 100 × *b*_∞_ for the standard sampling scheme applied to the original study reach and for the standard and modified sampling schemes applied to the expanded study reach. Parameter values are *p*_0_ = 0.1, 0.3, 0.5 and *q*_0_ = 0.1, 0.2, 0.3, 0.5, 0.7, 0.9. Note that *n*_crit_ is less than 150 for the standard sampling scheme applied to the original study reach but that the asymptotic relative bias is substantial: roughly 11% for *p*_0_ = 0.1, 43% for *p*_0_ = 0.3, and 100% for *p*_0_ = 0.5. *n*_crit_ is also less than 150 for both sampling schemes applied to the expanded study reach when the capture probability for sample 2 is at least 0.2, but the asymptotic relative biases are substantially reduced compared to those for the original study reach and are lower for the modified scheme (in fact, zero) than for the standard scheme. These results suggest that the asymptotic relative bias is a reasonably good estimate of the actual relative bias if the number of fish per zone is roughly 150 or more and the capture probability for sample 2 is at least 0.2. In such cases, the relative bias of Chapman’s estimator is substantially lower with the expanded study reach and the same level of sampling effort, regardless of whether the standard or modified sampling scheme is used. However, the estimator remains biased with the standard scheme but is unbiased for the modified scheme. For sample-2 capture probabilities of 0.1 or less, the asymptotic relative biases are unlikely to give acceptable approximations to the exact finite-*n* values unless the abundance of fish in each zone of the stream reach is much greater than 150 (≥ 500, say).

**Table 2:** Minimum sufficient abundances and asymptotic relative biases.

*p*_0_	*q*_0_	Standard, original				Standard, expanded				Modified, expanded			
		*q*	*q*′	*n*_crit_	PRB_∞_	*q*	*q*′	*n*_crit_	PRB_∞_	*q*	*q*′	*n*_crit_	PRB_∞_
0.1	0.100	0.271	0.271	62	11.1	0.100	0.100	458	3.4	0.271	0.100	482	0.0
	0.200	0.488	0.488	16	11.1	0.200	0.200	108	3.4	0.488	0.200	124	0.0
	0.300	0.657	0.657	7	11.1	0.300	0.300	45	3.4	0.657	0.300	56	0.0
	0.500	0.875	0.875	3	11.1	0.500	0.500	13	3.4	0.875	0.500	19	0.0
	0.700	0.973	0.973	1	11.1	0.700	0.700	5	3.4	0.973	0.700	8	0.0
	0.900	0.999	0.999	1	11.1	0.900	0.900	2	3.4	0.999	0.900	3	0.0
0.3	0.100	0.271	0.271	87	42.9	0.100	0.100	502	11.1	0.271	0.100	482	0.0
	0.200	0.488	0.488	24	42.9	0.200	0.200	119	11.1	0.488	0.200	124	0.0
	0.300	0.657	0.657	11	42.9	0.300	0.300	50	11.1	0.657	0.300	56	0.0
	0.500	0.875	0.875	5	42.9	0.500	0.500	15	11.1	0.875	0.500	19	0.0
	0.700	0.973	0.973	3	42.9	0.700	0.700	6	11.1	0.973	0.700	8	0.0
	0.900	0.999	0.999	3	42.9	0.900	0.900	2	11.1	0.999	0.900	3	0.0
0.5	0.100	0.271	0.271	135	100.0	0.100	0.100	553	20.0	0.271	0.100	482	0.0
	0.200	0.488	0.488	38	100.0	0.200	0.200	132	20.0	0.488	0.200	124	0.0
	0.300	0.657	0.657	19	100.0	0.300	0.300	55	20.0	0.657	0.300	56	0.0
	0.500	0.875	0.875	9	100.0	0.500	0.500	17	20.0	0.875	0.500	19	0.0
	0.700	0.973	0.973	7	100.0	0.700	0.700	7	20.0	0.973	0.700	8	0.0
	0.900	0.999	0.999	6	100.0	0.900	0.900	3	20.0	0.999	0.900	3	0.0

Minimum study-reach abundance (*n*_crit_) for which the percent relative bias of Chapman’s estimator is within 1% of the asymptotic percent relative bias (PRB_∞_). For each of three values of the probability *p*_0_ of exiting a single zone between samples 1 and 2, results are shown for the standard sampling scheme applied to the original study reach and for the standard and modified schemes applied to an expanded study reach three times as long, based on the same model of abundance and dispersal employed in Table 1. Other symbols are defined as in Table 1.

## Simulation study

Simulation studies of abundance estimators are useful for assessing the robustness of analytical results to simplifying assumptions invoked in their derivation and for documenting properties for which analytical results are not available [35]. Our analytical results are based on models where a fish’s dispersal probability is determined solely by the stream zone in which it is located when sample 1 is taken, without regard to its distance from the zone boundary. To assess the robustness of our main results to this simplifying assumption, we developed a spatially explicit simulation model in which each fish’s initial (sample 1) location in the stream is specified, and its position for sample 2 is determined by sampling from a probability distribution for net longitudinal movement distance. Whether a fish enters or exits the study reach between samples is implied by its locations for samples 1 and 2 relative to boundaries of the study reach. The simulation results also document the joint effects of dispersal and sampling variation on the precision of Chapman’s estimator, for which we do not have analytical results.

### Methods

Simulations were performed using the R programming language and computing environment, version 3.1.0 [36], with packages doParallel [37] and doRNG [38]. The main steps of the simulation for each combination of parameter values are as follows:

Specify habitat and fish parameters

Specify the number n.iter of iterations

Iterate n.iter times {

 Assign spatial locations to all fish

 Take first random sample and identify fish

 Allow all fish to move

 Take second random sample and identify fish

 Determine number of recaptures

 Calculate Chapman abundance estimate

}

Calculate empirical mean and bias of Chapman estimates

Calculate theoretical mean and bias of Chapman’s estimator.

These simulations build and expand upon those reported by Ruetz *et al.* [12].

Simulations were designed to shed light on two basic questions: (1) how effective is expanding the study reach as a means of decreasing the effect of dispersal on bias of Chapman’s estimator, and (2) does employing the modified sampling scheme instead of the standard scheme significantly increase the effectiveness of expanding the study reach? Most of the simulations addressed the second question. We describe methods for these simulations first, then indicate the minor changes made to address the first question.

In simulations comparing the standard and modified sampling schemes on an expanded study reach, the habitat comprised a study reach bordered by two buffer reaches: one upstream (zone US) and the other downstream (zone DS). Consistent with typical applications in stream fisheries management, the goal was to estimate fish abundance in the study reach, not total population size. The two buffer reaches allowed fish to move in and out of the study reach between samples 1 and 2. To assess the modified sampling scheme, the study reach was subdivided longitudinally into three subreaches of equal length (zones U, C, and D). The only important habitat parameters were the lengths of the study and buffer reaches and, for the modified sampling scheme, the lengths of the three zones within the study reach. The length of the study reach was set to 90 (arbitrary units), and the lengths of zones U, C, and D were set to 30 each. Lengths of both buffer reaches were set to 90. Fish parameters were the initial number of fish in each reach and subreach, capture probabilities for samples 1 and 2, and parameters specifying the maximum movement distance and the shape of the probability distribution for fish movement distances. The number of iterations was set to 10,000.

Initial longitudinal locations of fish in each stream zone were assigned by generating uniformly distributed pseudorandom variates. We used 75, 300, and 600 fish in the study reach (25, 100, and 200 fish in each zone). In model runs where balanced dispersal was desired (equal inward and outward dispersal, on average), we set the initial densities of fish in the two buffer reaches to be the same as in the study reach. Stochastic isotropic movement of all fish then resulted in approximately equal numbers of fish entering and exiting the study reach when averaged over the 10,000 iterations. When unbalanced dispersal was desired, we set the densities of fish in the two buffer reaches higher or lower than in the study reach so that inward dispersal was correspondingly higher or lower than outward dispersal, on average.

Determining which fish were captured in a given sample was accomplished by generating a Bernoulli pseudorandom variable for each fish, with probability of success (capture) equal to the specified capture probability. For the standard sampling scheme applied to zones U, C, and D, we assessed capture probabilities of *q* = *q*′ = 0.1, 0.3, 0.5, and 0.7. For the modified sampling scheme, sample 2 was taken from the entire study reach and had the same capture probabilities as for the standard scheme. Sample 1, however, was taken only from zone C, which is 1/3 the size of the total study reach. To make comparisons fair, we set the capture probability for sample 1 of the modified scheme so the duration of sampling was the same (instead of 1/3 as long) as for sample 2, based on Eq (11) (examples for the modified sampling scheme with equal capture probabilities for samples 1 and 2 are included in the supporting information (S2 File)).

As in Ruetz *et al.* [12], the longitudinal distance moved by each fish between samples 1 and 2 was determined by generating a pseudorandom variable from a symmetric beta distribution with both shape parameters set to 4, the mode shifted to 0, and the support stretched to [−*δ*, *δ*], where *δ* is the maximum longitudinal movement distance (values: 0, 30, 60, or 90). With *δ* = 90, an average of about 30% of fish in the study reach for sample 1 exited the reach before sample 2 was taken. Since the length of each zone of the study reach was 30, outward dispersal from the study reach was possible for at least some of the fish in the central zone when the maximum movement distance was greater than 30 but not otherwise.

Abundance in the study reach was estimated for each iteration using Chapman’s estimator. For both the standard and modified sampling schemes, simulations generated 10,000 abundance estimates for each of 48 combinations of true initial abundance (3 values), capture probability (4 values), and movement range (4 values). The empirical mean ${\bar{\hat{N}}}^{*}$ of Chapman’s estimator was calculated and compared with the theoretical value given by Eqs (8) or (10), the empirical relative biases $\bar{b}=\left({\bar{\hat{N}}}^{*}-n\right)/n$ and ${\bar{b}}^{\prime }=\left({\bar{\hat{N}}}^{*}-{\bar{N}}^{\prime }\right)/{\bar{N}}^{\prime }$ with respect to true abundance *n* and empirical mean abundance ${\bar{N}}^{\prime }$ when sample 2 was taken were calculated and compared, and the empirical interquartile range was determined. Since there were no simulation parameters corresponding to exit probabilities *p*, *p*_U_, *p*_C_, and *p*_D_ in the analytical results, values for these parameters were obtained by determining the average proportions of individuals that exited the corresponding parts of the study reach in the simulations.

In simulations comparing bias of abundance estimates based on sampling the original versus expanded study reaches, the length of the original study reach (zone C) was set to 30 and initial abundance was set to 120 fish. The length of the expanded study reach (zones U, C, and D) was set to 90 and initial abundance was set to 360, with 120 fish each in zone. Only the standard sampling scheme was applied to the original study reach. To make the duration of sampling the same as in other cases, we set the capture probabilities for samples 1 and 2 to the same values used for sample 1 (zone C) with the modified scheme applied to the expanded study reach.

### Results

**Fig 3:**
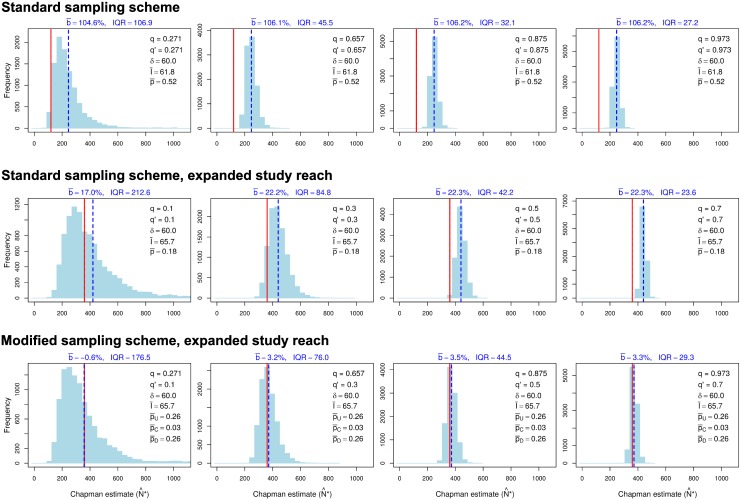
Effects of expanding the study reach on the empirical distribution of Chapman’s estimator with constant sampling effort. Top row: standard sampling scheme applied to a study reach of length 30 (arbitrary units) and abundance *n* = 120. Middle row: standard sampling scheme applied to an expanded study reach of length 90 and abundance *n* = 360. Bottom row: modified sampling scheme applied to an expanded study reach of length 90 and abundance *n* = 360, with 120 fish in each of zones U, C, and D. Sampling effort (duration of sampling) is the same in all cases, resulting in higher capture probabilities when sampling is restricted to a single zone. Maximum movement distance *δ* = 60 in all cases. *q*: sample-1 capture probability; *q*′: sample-2 capture probability; $\bar{p}$: average proportion of individuals in the study reach that exited between samples (standard sampling scheme only); ${\bar{p}}_{U}$, ${\bar{p}}_{C}$, ${\bar{p}}_{D}$: average proportion of individuals in zones U, C, and D of the study reach that exited the study reach between samples (modified sampling scheme only); $\bar{I}$: average number of immigrants in simulations; $\bar{b}$: average relative bias with respect to *n* (percent) of Chapman’s estimator in simulations; IQR: empirical interquartile range. Solid red lines: *n*; dashed blue lines: empirical mean of ${\hat{N}}^{*}$. See text for additional explanation.

We begin with results illustrating the efficacy of expanding the study reach to reduce dispersal-related bias. Fig 3 shows the empirical distribution of Chapman’s estimator for three cases, all with the same sampling effort and maximum dispersal distance *δ* = 60: (1) a study reach of length 30 with *n* = 120, sampled with the standard sampling scheme (top row), (2) an expanded study reach of length 90 with *n* = 360, sampled with the standard scheme (middle row), and (3) an expanded study reach of length 90 with *n* = 360, sampled with the modified scheme (bottom row). Consistent with the purpose of the modified scheme, the central zone is long enough so few fish located there when sample 1 is taken leave the study reach before sample 2 is taken (${\bar{p}}_{C}\approx 0.03$). Note that the relative bias of Chapman’s estimator for the original (short) study reach and standard sampling scheme consistently exceeds 100%. When the study reach is expanded, the relative bias drops to roughly 20% if the standard sampling scheme is used and to roughly 3% if the modified sampling scheme is used. This numerical example, and others with different choices of parameter values we have examined, confirm the pattern evident in Table 1: expanding the study reach is an effective way to reduce the effect of dispersal on the bias of Chapman’s estimator, even if the standard sampling scheme is retained, but bias typically is lower with the modified sampling scheme.

**Fig 4:**
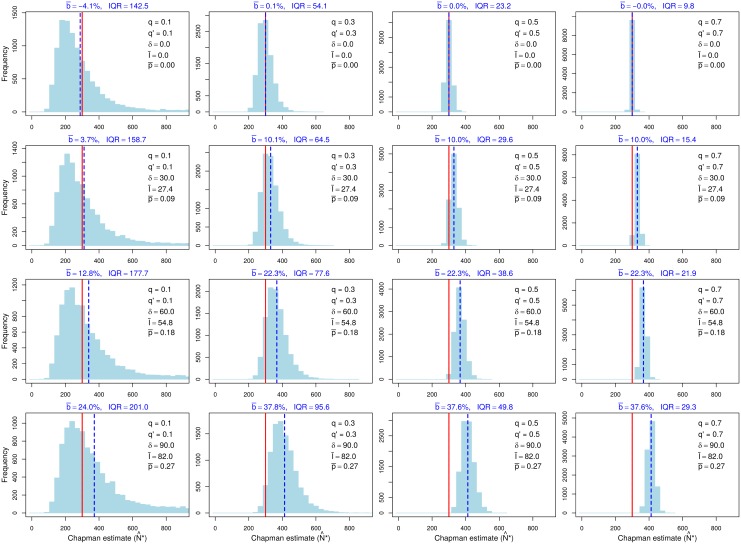
Empirical distribution of Chapman’s estimator in simulations with the standard sampling scheme. Study-reach length is 90 (arbitrary units), study-reach abundance *n* = 300, sample-1 capture probability *q* = 0.1, 0.3, 0.5, 0.7, sample-2 capture probability *q*′ = *q*, and maximum movement distance *δ* = 0, 30, 60, 90. Dispersal is balanced, so the average change in abundance between samples is approximately zero. Symbols and lines are defined as in Fig 3.

The remaining results compare efficacies of the standard and modified sampling schemes on the expanded study reach. Recall that the results in Table 1 show that the modified scheme typically is more effective than the standard scheme in reducing the bias of Chapman’s estimator when nonnegligible dispersal occurs. Simulation results for the standard scheme with dispersal (Tables 3 and 4) confirm the conclusion based on our analytical results that when sampling variation is taken into account, the relative bias of Chapman’s estimator can be positive or negative, with negative bias being particularly pronounced when capture probabilities are so low that few recaptures are made. Moderate to pronounced positive bias becomes common when the maximum movement distance is sufficiently long (i.e., dispersal across the study-reach boundary is sufficiently common), especially when capture probabilities are moderate to high and dispersal is either balanced (Table 3, Fig 4) or unbalanced in favor of inward dispersal (Table 4). When dispersal is unbalanced in favor of inward dispersal, bias tends to be more pronounced with respect to *n* than with respect to the expected value of *N*′, whereas the opposite pattern holds but when the imbalance favors outward dispersal (Table 4); Seber’s large-population, large-sample approximation in Eq (2) exhibits similar properties.

**Table 3:** Simulation results with balanced migration.

*n*	*δ*	*q*_0_	Standard sampling scheme											Modified sampling scheme												
			*q*	*q*′	$\bar{p}$	$\bar{I}$	${\bar{N}}^{\prime }$	${\bar{R}}^{\prime }$	${\bar{\hat{N}}}^{*}$	$E({\hat{N}}^{*})$	$\bar{b}$	${\bar{b}}^{\prime }$	IQR	*q*	*q*′	${\bar{p}}_{U}$	${\bar{p}}_{C}$	${\bar{p}}_{D}$	$\bar{I}$	${\bar{N}}^{\prime }$	${\bar{R}}^{\prime }$	${\bar{\hat{N}}}^{*}$	$E({\hat{N}}^{*})$	$\bar{b}$	${\bar{b}}^{\prime }$	IQR
75	0	0.1	0.1	0.1	0.0	0.0	75.0	0.8	46.0	46.1	-38.6	-38.6	30.0	0.27	0.1	0.0	0.0	0.0	0.0	75.0	0.7	43.7	43.9	-41.7	-41.7	28.0
		0.3	0.3	0.3	0.0	0.0	75.0	6.7	75.2	75.0	0.2	0.2	23.0	0.66	0.3	0.0	0.0	0.0	0.0	75.0	4.9	75.4	74.8	0.6	0.6	27.4
		0.5	0.5	0.5	0.0	0.0	75.0	18.7	75.0	75.0	0.0	0.0	11.1	0.88	0.5	0.0	0.0	0.0	0.0	75.0	10.9	75.3	75.0	0.4	0.4	17.1
		0.7	0.7	0.7	0.0	0.0	75.0	36.8	75.0	75.0	-0.1	-0.1	4.9	0.97	0.7	0.0	0.0	0.0	0.0	75.0	17.0	75.1	75.0	0.1	0.1	10.7
	30	0.1	0.1	0.1	0.1	6.8	75.0	0.7	47.8	47.8	-36.2	-36.2	33.0	0.27	0.1	0.1	0.0	0.1	6.8	75.0	0.7	43.9	43.8	-41.5	-41.4	28.0
		0.3	0.3	0.3	0.1	6.8	75.0	6.1	82.8	82.4	10.4	10.5	27.8	0.66	0.3	0.1	0.0	0.1	6.8	75.0	4.9	75.5	74.8	0.7	0.8	28.5
		0.5	0.5	0.5	0.1	6.8	75.0	17.0	82.5	82.5	10.0	10.1	14.4	0.88	0.5	0.1	0.0	0.1	6.8	75.0	10.9	75.2	75.0	0.3	0.4	17.6
		0.7	0.7	0.7	0.1	6.8	75.0	33.4	82.5	82.5	10.0	10.0	7.6	0.97	0.7	0.1	0.0	0.1	6.8	75.0	17.0	75.0	75.0	0.1	0.1	11.4
	60	0.1	0.1	0.1	0.2	13.6	74.9	0.6	49.5	49.6	-34.0	-33.9	32.5	0.27	0.1	0.3	0.0	0.3	13.6	74.9	0.7	44.3	44.3	-40.9	-40.8	28.0
		0.3	0.3	0.3	0.2	13.6	74.9	5.5	92.1	91.5	22.8	23.0	33.6	0.66	0.3	0.3	0.0	0.3	13.6	74.9	4.7	77.9	77.1	3.8	4.0	29.8
		0.5	0.5	0.5	0.2	13.6	74.9	15.3	91.6	91.6	22.2	22.4	19.2	0.88	0.5	0.3	0.0	0.3	13.6	74.9	10.5	77.6	77.4	3.5	3.7	18.7
		0.7	0.7	0.7	0.2	13.6	74.9	30.1	91.6	91.6	22.1	22.3	10.6	0.97	0.7	0.3	0.0	0.3	13.6	74.9	16.5	77.4	77.4	3.3	3.4	12.9
300	0	0.1	0.1	0.1	0.0	0.0	300.0	3.0	287.8	288.0	-4.1	-4.1	142.5	0.27	0.1	0.0	0.0	0.0	0.0	300.0	2.7	287.2	284.1	-4.3	-4.3	143.4
		0.3	0.3	0.3	0.0	0.0	300.0	27.0	300.3	300.0	0.1	0.1	54.1	0.66	0.3	0.0	0.0	0.0	0.0	300.0	19.7	300.4	300.0	0.1	0.1	64.3
		0.5	0.5	0.5	0.0	0.0	300.0	75.1	300.0	300.0	0.0	0.0	23.2	0.88	0.5	0.0	0.0	0.0	0.0	300.0	43.8	300.1	300.0	0.0	0.0	35.6
		0.7	0.7	0.7	0.0	0.0	300.0	147.2	299.9	300.0	0.0	0.0	9.8	0.97	0.7	0.0	0.0	0.0	0.0	300.0	68.1	300.0	300.0	0.0	0.0	21.9
	30	0.1	0.1	0.1	0.1	27.4	300.0	2.7	311.1	312.3	3.7	3.7	158.7	0.27	0.1	0.1	0.0	0.1	27.4	300.0	2.7	287.1	284.1	-4.3	-4.3	145.1
		0.3	0.3	0.3	0.1	27.4	300.0	24.6	330.4	330.1	10.1	10.1	64.5	0.66	0.3	0.1	0.0	0.1	27.4	300.0	19.7	300.2	300.0	0.1	0.1	65.5
		0.5	0.5	0.5	0.1	27.4	300.0	68.2	330.1	330.1	10.0	10.0	29.6	0.88	0.5	0.1	0.0	0.1	27.4	300.0	43.8	300.0	300.0	0.0	0.0	37.2
		0.7	0.7	0.7	0.1	27.4	300.0	133.7	330.1	330.1	10.0	10.0	15.4	0.97	0.7	0.1	0.0	0.1	27.4	300.0	68.1	300.0	300.0	0.0	0.0	24.2
	60	0.1	0.1	0.1	0.2	54.8	300.0	2.5	338.4	340.4	12.8	12.8	177.7	0.27	0.1	0.3	0.0	0.3	54.8	300.0	2.6	294.6	291.9	-1.8	-1.8	149.3
		0.3	0.3	0.3	0.2	54.8	300.0	22.1	366.8	367.0	22.3	22.3	77.6	0.66	0.3	0.3	0.0	0.3	54.8	300.0	19.1	310.2	309.9	3.4	3.4	69.7
		0.5	0.5	0.5	0.2	54.8	300.0	61.4	366.8	367.0	22.3	22.3	38.6	0.88	0.5	0.3	0.0	0.3	54.8	300.0	42.4	309.8	309.9	3.3	3.2	40.1
		0.7	0.7	0.7	0.2	54.8	300.0	120.3	367.0	367.0	22.3	22.3	21.9	0.97	0.7	0.3	0.0	0.3	54.8	300.0	66.0	309.8	309.9	3.3	3.3	27.0
600	0	0.1	0.1	0.1	0.0	0.0	600.0	6.0	601.2	598.8	0.2	0.2	244.0	0.27	0.1	0.0	0.0	0.0	0.0	600.0	5.4	598.9	598.0	-0.2	-0.2	252.3
		0.3	0.3	0.3	0.0	0.0	600.0	53.9	601.1	600.0	0.2	0.2	75.8	0.66	0.3	0.0	0.0	0.0	0.0	600.0	39.4	599.8	600.0	0.0	0.0	93.7
		0.5	0.5	0.5	0.0	0.0	600.0	149.9	600.3	600.0	0.0	0.0	33.3	0.88	0.5	0.0	0.0	0.0	0.0	600.0	87.5	600.3	600.0	0.0	0.0	51.5
		0.7	0.7	0.7	0.0	0.0	600.0	293.9	600.0	600.0	0.0	0.0	14.3	0.97	0.7	0.0	0.0	0.0	0.0	600.0	136.3	599.9	600.0	0.0	0.0	31.3
	30	0.1	0.1	0.1	0.1	54.8	600.1	5.4	661.0	658.0	10.2	10.1	279.6	0.27	0.1	0.1	0.0	0.1	54.8	600.1	5.4	599.4	598.1	-0.1	-0.1	252.4
		0.3	0.3	0.3	0.1	54.8	600.1	48.9	662.0	660.3	10.3	10.3	90.9	0.66	0.3	0.1	0.0	0.1	54.8	600.1	39.4	600.3	600.1	0.0	0.0	94.6
		0.5	0.5	0.5	0.1	54.8	600.1	136.2	660.6	660.3	10.1	10.1	42.3	0.88	0.5	0.1	0.0	0.1	54.8	600.1	87.5	600.4	600.1	0.1	0.1	53.0
		0.7	0.7	0.7	0.1	54.8	600.1	267.1	660.3	660.3	10.0	10.0	22.0	0.97	0.7	0.1	0.0	0.1	54.8	600.1	136.3	599.9	600.1	0.0	0.0	34.1
	60	0.1	0.1	0.1	0.2	109.5	600.2	4.9	733.6	729.4	22.3	22.2	325.3	0.27	0.1	0.3	0.0	0.3	109.5	600.2	5.2	617.4	617.4	2.9	2.9	262.1
		0.3	0.3	0.3	0.2	109.5	600.2	44.0	735.9	734.0	22.6	22.6	111.5	0.66	0.3	0.3	0.0	0.3	109.5	600.2	38.2	620.0	619.9	3.3	3.3	101.6
		0.5	0.5	0.5	0.2	109.5	600.2	122.5	734.3	734.0	22.4	22.3	54.3	0.88	0.5	0.3	0.0	0.3	109.5	600.2	84.7	620.2	619.9	3.4	3.3	57.7
		0.7	0.7	0.7	0.2	109.5	600.2	240.3	734.0	734.0	22.3	22.3	31.0	0.97	0.7	0.3	0.0	0.3	109.5	600.2	131.9	619.7	619.9	3.3	3.3	38.4

Results for selected choices of true initial abundance *n*, maximum longitudinal movement distance *δ* (arbitrary units), and capture probabilities *q* amd *q*′. Capture probabilities for sample 1 of the modified scheme are adjusted so sampling effort is the same as for sample 2. $\bar{p}$: average proportion of individuals exiting the study reach between samples (standard sampling scheme); ${\bar{p}}_{U}$, ${\bar{p}}_{C}$, ${\bar{p}}_{D}$: average proportion of individuals in zones U, C, and D of the study reach for sample 1 that exited the study reach between samples (modified scheme); $\bar{I}$: average number of individuals entering the study reach between samples; $\bar{N^{\prime }}$: average true abundance when sample 2 was taken; ${\bar{R}}^{\prime }$: average number of recaptures; ${\bar{\hat{N}}}^{*}$: average value of ${\hat{N}}^{*}$ in simulations; $E({\hat{N}}^{*})$: theoretical unconditional expected value of ${\hat{N}}^{*}$; $\bar{b}$: percent relative bias of ${\hat{N}}^{*}$ with respect to *n* in simulations; ${\bar{b}}^{\prime }$: percent relative bias of ${\hat{N}}^{*}$ with respect to ${\bar{N}}^{\prime }$ in simulations; IQR: interquartile range (3rd minus 1st quartile) of ${\hat{N}}^{*}$ in simulations.

**Table 4:** Simulation results with unbalanced migration.

B:S	*δ*	*q*_0_	Standard sampling scheme											Modified sampling scheme												
			*q*	*q*′	$\bar{p}$	$\bar{I}$	${\bar{N}}^{\prime }$	${\bar{R}}^{\prime }$	${\bar{\hat{N}}}^{*}$	$E({\hat{N}}^{*})$	$\bar{b}$	${\bar{b}}^{\prime }$	IQR	*q*	*q*′	${\bar{p}}_{U}$	${\bar{p}}_{C}$	${\bar{p}}_{D}$	$\bar{I}$	${\bar{N}}^{\prime }$	${\bar{R}}^{\prime }$	${\bar{\hat{N}}}^{*}$	$E({\hat{N}}^{*})$	$\bar{b}$	${\bar{b}}^{\prime }$	IQR
1.5	0	0.1	0.1	0.1	0.0	0.0	300.0	3.0	289.0	288.0	-3.7	-3.7	143.6	0.27	0.1	0.0	0.0	0.0	0.0	300.0	2.7	282.2	284.1	-5.9	-5.9	142.7
		0.3	0.3	0.3	0.0	0.0	300.0	27.0	299.7	300.0	-0.1	-0.1	52.5	0.66	0.3	0.0	0.0	0.0	0.0	300.0	19.7	299.1	300.0	-0.3	-0.3	63.8
		0.5	0.5	0.5	0.0	0.0	300.0	74.9	300.0	300.0	0.0	0.0	23.3	0.88	0.5	0.0	0.0	0.0	0.0	300.0	43.8	299.7	300.0	-0.1	-0.1	35.6
		0.7	0.7	0.7	0.0	0.0	300.0	146.9	300.0	300.0	0.0	0.0	10.1	0.97	0.7	0.0	0.0	0.0	0.0	300.0	68.1	299.9	300.0	0.0	0.0	21.5
	30	0.1	0.1	0.1	0.1	40.9	313.6	2.7	326.8	326.4	8.9	4.2	168.7	0.27	0.1	0.1	0.0	0.1	40.9	313.6	2.7	295.3	297.0	-1.6	-5.8	150.0
		0.3	0.3	0.3	0.1	40.9	313.6	24.5	344.7	345.0	14.9	9.9	66.6	0.66	0.3	0.1	0.0	0.1	40.9	313.6	19.7	312.9	313.6	4.3	-0.2	67.9
		0.5	0.5	0.5	0.1	40.9	313.6	68.1	345.0	345.0	15.0	10.0	32.6	0.88	0.5	0.1	0.0	0.1	40.9	313.6	43.8	313.3	313.6	4.4	-0.1	38.7
		0.7	0.7	0.7	0.1	40.9	313.6	133.5	345.0	345.0	15.0	10.0	17.4	0.97	0.7	0.1	0.0	0.1	40.9	313.6	68.1	313.5	313.6	4.5	0.0	25.3
	60	0.1	0.1	0.1	0.2	81.9	327.2	2.4	372.3	371.1	24.1	13.8	198.4	0.27	0.1	0.3	0.0	0.3	81.9	327.2	2.6	316.2	318.3	5.4	-3.4	160.5
		0.3	0.3	0.3	0.2	81.9	327.2	22.1	400.0	400.2	33.3	22.2	84.3	0.66	0.3	0.3	0.0	0.3	81.9	327.2	19.1	337.7	338.1	12.6	3.2	76.3
		0.5	0.5	0.5	0.2	81.9	327.2	61.3	400.4	400.2	33.5	22.3	42.4	0.88	0.5	0.3	0.0	0.3	81.9	327.2	42.4	338.1	338.1	12.7	3.3	45.0
		0.7	0.7	0.7	0.2	81.9	327.2	120.1	400.2	400.2	33.4	22.3	24.8	0.97	0.7	0.3	0.0	0.3	81.9	327.2	65.9	338.1	338.1	12.7	3.3	30.5
0.5	0	0.1	0.1	0.1	0.0	0.0	300.0	3.0	288.5	288.0	-3.8	-3.8	139.5	0.27	0.1	0.0	0.0	0.0	0.0	300.0	2.7	281.2	284.1	-6.3	-6.3	140.0
		0.3	0.3	0.3	0.0	0.0	300.0	27.0	300.4	300.0	0.1	0.1	52.0	0.66	0.3	0.0	0.0	0.0	0.0	300.0	19.8	299.6	300.0	-0.1	-0.1	64.8
		0.5	0.5	0.5	0.0	0.0	300.0	75.0	300.0	300.0	0.0	0.0	23.2	0.88	0.5	0.0	0.0	0.0	0.0	300.0	43.8	299.8	300.0	-0.1	-0.1	35.4
		0.7	0.7	0.7	0.0	0.0	300.0	147.0	300.0	300.0	0.0	0.0	10.0	0.97	0.7	0.0	0.0	0.0	0.0	300.0	68.2	299.9	300.0	0.0	0.0	21.8
	30	0.1	0.1	0.1	0.1	13.6	286.3	2.7	298.0	298.1	-0.7	4.1	149.6	0.27	0.1	0.1	0.0	0.1	13.6	286.3	2.7	268.2	271.3	-10.6	-6.3	133.3
		0.3	0.3	0.3	0.1	13.6	286.3	24.5	315.4	315.0	5.1	10.2	59.2	0.66	0.3	0.1	0.0	0.1	13.6	286.3	19.8	286.0	286.3	-4.7	-0.1	61.7
		0.5	0.5	0.5	0.1	13.6	286.3	68.2	315.1	315.0	5.0	10.0	27.4	0.88	0.5	0.1	0.0	0.1	13.6	286.3	43.8	286.1	286.3	-4.6	-0.1	34.3
		0.7	0.7	0.7	0.1	13.6	286.3	133.7	315.0	315.0	5.0	10.0	13.7	0.97	0.7	0.1	0.0	0.1	13.6	286.3	68.2	286.3	286.3	-4.6	0.0	22.5
	60	0.1	0.1	0.1	0.2	27.2	272.5	2.5	309.3	309.4	3.1	13.5	164.5	0.27	0.1	0.3	0.0	0.3	27.2	272.5	2.6	262.4	265.3	-12.5	-3.7	133.5
		0.3	0.3	0.3	0.2	27.2	272.5	22.1	333.5	333.3	11.2	22.4	66.9	0.66	0.3	0.3	0.0	0.3	27.2	272.5	19.1	281.1	281.5	-6.3	3.1	62.9
		0.5	0.5	0.5	0.2	27.2	272.5	61.4	333.2	333.3	11.1	22.2	33.4	0.88	0.5	0.3	0.0	0.3	27.2	272.5	42.4	281.3	281.5	-6.2	3.2	35.5
		0.7	0.7	0.7	0.2	27.2	272.5	120.3	333.1	333.3	11.0	22.2	18.1	0.97	0.7	0.3	0.0	0.3	27.2	272.5	66.0	281.5	281.5	-6.2	3.3	23.7

Simulation results corresponding to those in Table 3 with *n* = 300 but with the initial number of individuals in the buffer zones increased or decreased so that average inward dispersal was greater or less than average outward dispersal. B:S is the ratio of initial density (number per unit area) of individuals in the buffer zones to the initial density in the study reach. Average dispersal is balanced when B:S = 1, as in Table 3. Inward dispersal exceeds outward on average when B:S > 1; the opposite occurs when B:S < 1. Other symbols are defined as in Table 3.

**Fig 5:**
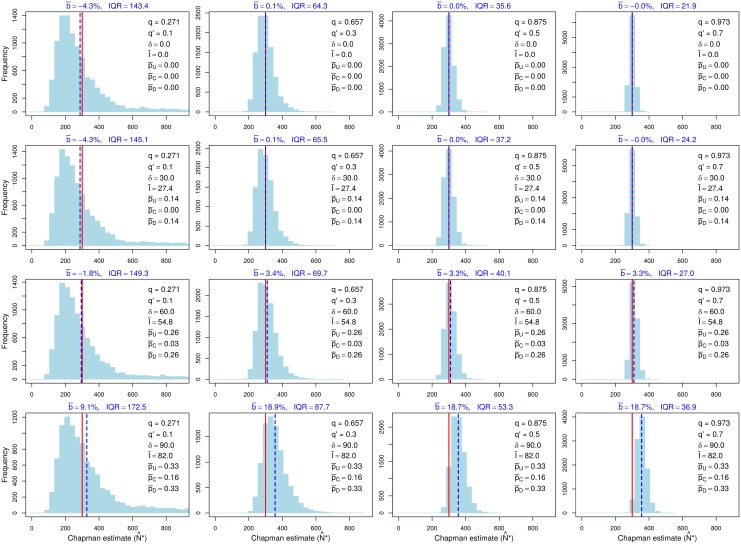
Empirical distribution of Chapman’s estimator in simulations with the modified sampling scheme. Study-reach length is 90 (arbitrary units), study-reach abundance *n* = 300, sample-2 capture probability *q*′ = 0.1, 0.3, 0.5, 0.7, sample-1 capture probability *q* = 1 − (1 − *q*′)^3^, and maximum movement distance *δ* = 0, 30, 60, 90. Sample-1 capture probabilities ensure sampling effort (duration of sampling) is the same as in sample 2. Dispersal is balanced. Other symbols and lines are defined as in Fig 3.

The effectiveness of the modified sampling scheme in reducing the effect of dispersal on bias can be seen in Tables 3 and 4 and in Figs 3 and 5. In all cases with dispersal where the capture probabilities are high enough so at least 4 recaptures were made on average, the relative bias of Chapman’s estimator is smaller with the modified sampling scheme than with the standard scheme, even with unbalanced dispersal.

The key role played by capture probabilities in determining precision is evident in all the tables and figures: increasing the capture probabilities (moving to the right in any row of Figs 3, 4, or 5) consistently increases precision. This is true even when there is a pronounced positive bias due to dispersal, in which case the entire empirical distribution of Chapman’s estimator may exceed the true population size (e.g., bottom right panel of Fig 4), virtually guaranteeing that a single estimate drawn from this distribution in a field study will be positively biased. Dispersal also affects precision, with increasing dispersal tending to decrease precision. This effect, however, is subtler than that of the capture probabilities over the range of values considered.

In all cases addressed in the simulations, the theoretical unconditional mean calculated using Eqs (8) or (10) is very close to the corresponding average value of the abundance estimator in the simulation, despite the difference in dispersal assumptions. The examples shown in Tables 3 and 4 illustrate this fact (compare the columns labeled ${\bar{\hat{N}}}^{*}$ and $E({\hat{N}}^{*})$). We interpret this result to indicate that the theoretical unconditional means are reasonably robust to the simplifying assumptions regarding dispersal on which they are based, regardless of which sampling scheme is used or whether dispersal is balanced or unbalanced.

Finally, we note that Tables 3 and 4, and Figs 3–5, include only a subset of the combinations of parameter values employed in the simulation study. Histograms for additional combinations of parameter values are provided in the supporting information (S2 File).

## Discussion

We presented and assessed expressions for the expected value and bias of Chapman’s estimator with dispersal that are valid for arbitrary study-reach abundances and properly account for the effect of sampling variation on bias. To the best of our knowledge, these are the first such expressions to appear in the literature on two-sample mark-recapture abundance estimation, which is one of the most commonly used methods of abundance estimation in stream fisheries management today. We also presented and assessed expressions for the expected value and bias of Chapman’s estimator for a new modification of the standard sampling scheme in which the study reach is expanded and fish for sample 1 are caught and released only in a central zone of the study reach so that few or no marked fish exit the study reach between samples 1 and 2. Our analytical and numerical results indicate that, compared to the standard sampling scheme with the same level of sampling effort, this new sampling scheme can substantially reduce or eliminate dispersal-related bias in Chapman’s estimator while still requiring only temporary batch marking of fish and two days in the field.

As abundance in the study reach becomes large, expressions for the mean and bias of Chapman’s estimator converge to asymptotic forms that agree with the corresponding expressions for the MRR, which assume large samples from a large population and therefore ignore the effects of sampling variation on bias. These asymptotic forms indicate that expanding the study reach and retaining the standard sampling scheme is an effective way to reduce dispersal-related bias in Chapman’s estimator, but that expanding the study reach and switching to the modified sampling scheme with the same level of sampling effort is typically even more effective if there is significant dispersal (exit probabilities of roughly 0.1 or greater). The modified sampling scheme is not intended for situations where there is no dispersal, and it is not more effective than the standard scheme in such cases.

Our numerical results suggest that when there is significant dispersal, the asymptotic expressions for the bias of Chapman’s estimator adequately approximate the exact finite-*n* expressions if abundance in the study reach is roughly 150 or more and capture probabilities for both samples are at least 0.2. Abundances of roughly 500 or more may be required if either capture probability is 0.1 or lower. Comparison of sampling schemes becomes more difficult when both study-reach abundance and capture probability are low (less than roughly 100 and 0.1, respectively), because bias then becomes rather sensitive to abundance, capture probability, and exit probability.

The bias expressions for finite *n* contain two terms: one mainly reflecting the effect of dispersal and the other the effect of sampling variation. The term reflecting the effect of sampling variation approaches zero as abundance becomes large, leading to the asymptotic expressions just mentioned. The minimum abundance required for these asymptotic expressions to closely approximate the exact relative bias is strongly dependent on the mark-recapture probability, which is the probability of any given fish being caught in both the first sample (and therefore marked) and the second sample (and therefore recaptured). This fact can be seen by noting that the term reflecting the effect of sampling variation in each bias expression includes a factor of form λ^*n*_*x*_−1^, where *n*_*x*_ is abundance in either the study reach or its central zone and λ = 1 − (mark-recapture probability). This term decays geometrically toward zero at a rate that decreases with decreasing mark-recapture probability: the smaller the mark-recapture probability, the closer λ will be to 1 and hence the more pronounced the effect of sampling variability on relative bias will be for any given abundance *n*_*x*_. In this connection, it is interesting to note that Robson and Regier [18], in their classic paper on 2-sample mark-recapture abundance estimation, noted that low numbers of recaptures (fewer than 7) tend to create pronounced negative bias in Chapman’s estimator when there is no dispersal.

A common method of reducing the influence of dispersal on bias of Chapman’s estimator in streams is to lengthen the study reach. Because it increases study-reach abundance and sample sizes, this approach also reduces the effect of sampling variation on bias. We proposed the modified sampling scheme as a way to further reduce the effect of dispersal on Chapman’s estimator with the same level of sampling effort. The study reach is lengthened by moving the upstream boundary further upstream and the downstream boundary further downstream. Fish for sample 1 are captured and released only in the central zone, which coincides with the original study reach, while sample 2 is taken from the entire expanded study reach. If the time between samples 1 and 2 is sufficiently short and the distance between boundaries of the expanded study reach and central zone is sufficiently large relative to fish movement rates, few if any marked fish will exit the study reach between samples. Dispersal will then have no meaningful effect on the relative bias of Chapman’s estimator with respect to the expected value E(*N*′) of abundance when sample 2 is taken. Sampling variation will sometimes remain a significant factor if abundance in the central zone is low (especially if capture probabilities are also low), reducing the mark-recapture probability and resulting in a negative bias with respect to E(*N*′). Based on numerical examples, this bias appears to become negligible when abundance in the central zone and the capture probabilities are jointly large enough so the expected value of the number of recaptures in sample 2 is roughly 4 or more (e.g., Tables 3 and 4).

A practical issue with the modified sampling scheme is how much to expand the study reach beyond its original boundaries. This problem is not unique to abundance estimation and has been considered in the design of mark-recapture studies of stream fish movement [39]. One would like the minimum distance between the boundaries of the original and expanded study reaches to exceed, say, the 95th percentile of the distribution of movement distances during a period of time equal to the inter-sample interval, but sufficient information about this distribution will rarely be available and would be costly to obtain. A more-pragmatic approach is to conduct a pilot mark-recapture experiment in which individuals are captured, marked, and released in the original study reach, and a second sample is taken from a reach extending upstream and downstream as far as feasible, with the distance of each recapture from the nearest boundary of the original study reach being recorded. The size of the expanded study reach is then chosen so the minimum distance between boundaries of the original and expanded study reaches is somewhat greater than the maximum observed recapture distance.

In closing, we wish to mention a few alternatives to the 2-sample mark-recapture method and why the newer, more-sophisticated methods are rarely used by stream fishery managers in routine abundance assessments. The MRR estimator came into widespread use by marine and freshwater fisheries scientists in northern Europe around 1900. While appropriate for many inland fisheries (especially streams), the closed-population assumptions on which this method is based are dubious for marine fisheries and many wildlife populations. Not surprisingly, then, a variety of more-sophisticated open-population methods have been developed—most of which were originally designed for application to wildlife populations—that can be applied even if significant recruitment, mortality, and dispersal occur (e.g., Jolly-Seber method, Pollock’s robust design, spatial mark-recapture [1, 4, 40, 41]). Since the biological assumptions of these methods are less restrictive, and free high-quality software is available that removes any technical barriers to their use, one might expect them to have been widely adopted by stream fishery managers. Why has this not occurred?

The advantages of open-population methods are purchased at a cost: they require permanent, unique marking of captured fish (instead of only temporary batch marking) and multiple site visits (instead of only one or two) so that individual capture histories can be constructed that are long enough to permit accurate and precise estimates of model parameters characterizing recruitment, mortality, and dispersal. In wildlife management applications, this added cost usually is minor because of the small number of populations that any given management agency must assess, and the use of open-population estimation methods is therefore becoming the norm. In stream fisheries, however, where abundance typically is assessed at the reach scale, the additional cost of labor and supplies to agencies responsible for managing inland fisheries is prohibitive because of the large number of reaches that must be assessed. Thus, although inland fisheries managers typically are aware of the more-sophisticated alternatives, the 2-sample mark-recapture method continues to be used far more commonly than any of the open-population methods for monitoring abundance in stream fisheries around the world, with use of open-population methods being largely restricted to special studies (e.g., [42]). Indeed, the main alternatives are single-pass electrofishing (catch per unit effort) and the 3-pass removal (depletion) method, which require no marking of fish and only a single site visit [13, 43–48].

Single-pass electrofishing is perhaps the simplest and quickest method of assessing fish abundance in streams. It yields estimates that are approximately proportional to abundance for fixed sampling effort and catchability [43–48]. These estimates of relative abundance are often expressed as catch per unit effort (CPUE, the number of captures divided by a measure of sampling effort) to reduce their dependence on effort and thus partially standardize them. CPUE estimates can be useful in documenting qualitative temporal trends in abundance, provided the factors affecting catchability (gear and settings, crew skill, habitat characteristics, etc.) are approximately constant across surveys, which in practice is difficult to ensure. The 3-pass removal method is capable of producing accurate estimates of absolute abundance but requires significantly more time and effort than single-pass electrofishing. Three-pass removal is not appropriate for large populations (because successive samples must exhibit depletion due to sampling), but this constraint often is not problematic in reach-scale assessments of streams. A more serious problem is that this method is prone to substantial negative bias unless the true capture probabilities in successive samples are consistently high (≥ 0.5) [11, 12, 29, 47, 49].

Because sampling for single-pass electrofishing and 3-pass removal is completed in a single day, these methods are thought to be less subject to dispersal-related bias than is the 2-sample mark-recapture method with the standard sampling scheme [13]. However, as just mentioned, both of these methods have significant weaknesses, especially if estimates of absolute abundance are required. If the 2-sample mark-recapture method with the standard sampling scheme is used instead, the effect of dispersal on the relative bias of Chapman’s estimator can be reduced by expanding the study reach. Another practical approach found to be effective for rainbow trout (*Oncorhynchus mykiss*) is to limit opportunities for dispersal by reducing the recovery period between samples to several hours [14]. The modified sampling scheme proposed in the present paper is another practical alternative for estimating absolute abundance that can reduce or eliminate dispersal-related bias while retaining the standard 1-day recovery period, though our assessment of it must remain tentative until a significant body of evidence is available from field applications. All of these pragmatic approaches to reducing dispersal effects on abundance estimates share the desirable properties of requiring only temporary batch marking of fish (or no marking at all) and no more than two days in the field.

## Supporting information

S1 File Derivation of analytical results. (PDF)

S2 File Simulation results. (PDF)
