# Supplementary material for: Reducing effects of dispersal on the bias of 2-sample mark-recapture estimators of stream fish abundance
Source: PLoS One. 2018 Aug 1;13(8):e0200733. doi: 10.1371/journal.pone.0200733 (PMC6070202; doi:10.1371/journal.pone.0200733)
Supplement: S1 File — (PDF) [file pone.0200733.s001.pdf]

Reducing effects of dispersal on the bias of 2-sample mark-recapture abundance estimators

James N. McNair[1], Carl R. Ruetz III[1], Ariana Carlson[1], and Jiyeon Suh[2]

[1]Annis Water Resources Institute, Grand Valley State University, 740 West Shoreline Drive, Muskegon, Michigan 49441 and [2]Department of Mathematics, Grand Valley State University, 1 Campus Drive, Allendale, Michigan 49401, USA

Supporting information 1: Derivation of analytical results

Here we outline derivations of the expected value of Chapman's abundance estimator for the standard sampling scheme with and without dispersal and for the modified sampling scheme with dispersal. We begin with the modified sampling scheme, since this case includes the others as special cases. Chapman's estimator is given by

$$\hat{N}^* = \frac{(C' + 1)(M + 1)}{R' + 1} - 1,$$

where M, C', and R' are discrete random variables representing the number of fish captured, marked, and released in sample 1 (M), the total number of fish captured in sample 2 (C'), and the number of marked fish recaptured in sample 2 (R'). To avoid unnecessarily complicating the theory, we assume that the probability of exiting the study reach between samples 1 and 2 is uniform for all fish in the study reach (standard sampling scheme) or in the same zone of the study reach (modified scheme), and we interpret this shared exit probability as the weighted average of spatially-explicit exit probabilities for the study reach or zone, respectively.

Expected value of Chapman's estimator with the modified sampling scheme

Recall from the text that when the modified sampling scheme is used, the study reach is divided longitudinally into three zones labeled U, C, and D, with U being the upstream zone, C the central zone, and D the downstream zone. Let n_U, n_C, and n_D be the numbers of fish in the three zones when sample 1 is taken, and let $n = n_U + n_C + n_D$ be the total number of fish in the study reach. These abundances are treated as parameters. Sample 1 is taken only from zone C, and all fish captured in this sample are returned to zone C after marking. Sample 2 is taken from the entire study reach.

Suppose we knew the values of M, C', and R'. Calling these values m, c', and r', the value of Chapman's estimator would also be known and would be given by $(c' + 1)(m + 1)/(r' + 1) - 1$. We are interested in what is essentially the opposite extreme, where no sampling has occurred yet, the values of M, C', and R' are all unknown, and we wish to find the expected value of Chapman's estimator, taking into account all possible values of M, C', and R'. The basic idea underlying our approach is to begin by assuming M, C', and R' are known and then successively remove conditioning on knowing the number of recaptures in sample 2, the total number of captures in sample 2, and so on.

Let O_m and O_u be random variables representing the number of marked and unmarked fish that exit the study area between samples 1 and 2, and let I_u be a random variable representing the number of fish that

enter. Let $E(\hat{N}^* \mid m, o_m, o_u, i_u, c')$ denote the expected value of Chapman's estimator, given $M = m$, $O_m = o_m$, $O_u = o_u$, $I_u = i_u$, and $C' = c'$, where the conditional expectation is taken over all possible values of R'. Then we can write

$$E(\hat{N}^* \mid m, o_m, o_u, i_u, c') = (c' + 1)(m + 1)\, E\left(\frac{1}{R' + 1} \,\middle|\, m, o_m, o_u, i_u, c'\right) - 1. \tag{1}$$

Assuming Bernoulli sampling, it is well known that the conditional distribution of the number R' of recaptures in sample 2 has the hypergeometric distribution given by

$$P\{R' = r' \mid m, o_m, o_u, i_u, c'\} = \binom{m'}{r'}\binom{n' - m'}{c' - r'} \Big/ \binom{n'}{c'}, \tag{2}$$

where $m' = m - o_m$ and $n' = n - o_m + i_u - o_u$ [1, 3]. A straightforward calculation using Eqs (1) and (2) yields

$$E(\hat{N}^* \mid m, o_m, o_u, i_u, c') = \begin{cases} m + (n' - m')\dfrac{m + 1}{m' + 1} - \dfrac{m + 1}{m' + 1}\,\dfrac{(n' - m')!(n' - c')!}{n'!(n' - m' - c' - 1)!}, & \text{if } c' < n' - m' \\ m + (n' - m')\dfrac{m + 1}{m' + 1}, & \text{if } c' \geqslant n' - m'. \end{cases} \tag{3}$$

Next we remove the conditioning on C'. Given the values o_m, o_u, and i_u of random variables O_m, O_u, and I_u, random variable C' has a binomial distribution with parameters n' and q'. Then taking the expected value of Eq (3) over all possible values of C', we find that

$$E(\hat{N}^* \mid m, o_m, o_u, i_u) = m + (n - m + i_u - o_u)\frac{m + 1}{m' + 1}\left[1 - (1 - q')^{m'+1}\right] \tag{4}$$

The next step is to remove the conditioning on I_u and O_u. I_u is assumed to be independent of the numbers of marked and unmarked fish in the study reach. O_u is assumed to be the sum of three binomial random variables: the numbers of unmarked fish exiting the three zones of the study reach. Letting p_U, p_C, and p_D be the exit probabilities for the three zones, the binomial parameters are n_U and p_U for zone U, $n_C - m$ and p_C for zone C, and n_D and p_D for zone D. Then assuming all fish enter and exit the study reach independently,

$$E(O_u \mid m, o_m) = n_U p_U + (n_C - m)p_C + n_U p_U = n\tilde{p} - m p_C, \tag{5}$$

where $\tilde{p} = (n_U/n)p_U + (n_C/n)p_C + (n_D/n)p_D$. Taking the expected value of Eq (4) and using Eq (5), we find that

$$E(\hat{N}^* \mid m, o_m) = m + \left[n(1 - \tilde{p}) + \tilde{I} - m(1 - p_C)\right]\frac{m + 1}{m' + 1}\left[1 - (1 - q')^{m'+1}\right], \tag{6}$$

where $\tilde{I} = E(I_u)$.

To remove the conditioning on O_m, we note that the number of marked fish exiting the study area (from zone C) between samples 1 and 2 is binomial with parameters m and p_C. Taking the expected value of Eq (6) and recalling that $m' = m - o_m$, a straightforward calculation yields

$$E(\hat{N}^* \mid m) = \frac{n(1 - \tilde{p}) + \tilde{I}}{1 - p_C} - \frac{n(1 - \tilde{p}) + \tilde{I}}{1 - p_C}\left[1 - q'(1 - p_C)\right]^{m+1} + m\left[1 - q'(1 - p_C)\right]^{m+1}. \tag{7}$$

Finally, we remove conditioning on M, which is binomial with parameters n_C and q. Taking the expected value of Eq (7), straightforward calculations yield

$$E(\hat{N}^*) = \Omega_2 - \varphi_2\, \lambda_2^{n_C - 1} \tag{8}$$

where

$$\begin{aligned}\Omega_2 &= \frac{n(1-\tilde{p})+\tilde{I}}{1-p_{\mathrm{C}}}\\ \varphi_2 &= \Big(\Omega_2[1-qq'(1-p_{\mathrm{C}})]-n_{\mathrm{C}}q[1-q'(1-p_{\mathrm{C}})]\Big)[1-q'(1-p_{\mathrm{C}})]\\ \lambda_2 &= 1-qq'(1-p_{\mathrm{C}}).\end{aligned}$$

This is Eq (10) of the text.

Standard sampling scheme with dispersal

The standard sampling scheme is a special case of the modified sampling scheme where all the fish are in zone C, which becomes the study reach. To obtain an expression for the expected value of Chapman's estimator in this case, we set $n_{\mathrm{U}} = 0 = n_{\mathrm{D}}$, implying $n_{\mathrm{C}} = n$ and $\tilde{p} = p_{\mathrm{C}} = p$. Then Eq (8) above becomes

$$\mathrm{E}(\hat{N}^*) = \Omega_1 - \varphi_1\,\lambda_1^{n-1} \tag{9}$$

where

$$\begin{aligned}\Omega_1 &= n+\frac{\tilde{I}}{1-p}\\ \varphi_1 &= \Big(\Omega_1[1-qq'(1-p)]-nq[1-q'(1-p)]\Big)[1-q'(1-p)]\\ \lambda_1 &= 1-qq'(1-p).\end{aligned}$$

This is Eq (8) of the text.

When comparing the standard and modified sampling schemes applied to the expanded study reach, it is important to ensure that the average probability of exiting the study reach between samples 1 and 2 is the same for both schemes. To do this, we choose $p = \tilde{p}$ in Eq (9). It is also important to ensure that sampling effort for each sample is the same for both sampling schemes. To do this, we require q' to be the same for both schemes, and in accordance with Eq (11) of the text, we require $q = 1-(1-q')^k$ for the modified scheme, where the size of the study area is k times the size of zone C.

Standard sampling scheme without dispersal

This case is the one considered by Skalski and Robson [2] and is simply the previous case with dispersal turned off. Setting $p = 0 = \tilde{I}$ in Eq (9), we find at once that

$$\mathrm{E}(\hat{N}^*) = \Omega_0 - \varphi_0\,\lambda_0^{n-1}$$

where

$$\begin{aligned}\Omega_0 &= n\\ \varphi_0 &= n(1-q)(1-q')\\ \lambda_0 &= 1-qq'.\end{aligned}$$

This is Eq (6) of the text.

References

[1] Chapman DG. Some properties of the hypergeometric distribution with applications to zoological sample censuses. University of California Publications in Statistics. 1951; 1:131–160.

[2] Skalski JR, Robson DS. Techniques for Wildlife Investigations: Design and Analysis of Capture Data. San Diego: Academic Press; 1992.

[3] Wittes JT. On the bias and estimated variance of Chapman's two-sample capture-recapture population estimate. Biometrics. 1972; 28:592–597.
