# Supplementary material for: Reducing effects of dispersal on the bias of 2-sample mark-recapture estimators of stream fish abundance
Source: PLoS One. 2018 Aug 1;13(8):e0200733. doi: 10.1371/journal.pone.0200733 (PMC6070202; doi:10.1371/journal.pone.0200733)
Supplement: S2 File — (PDF) [file pone.0200733.s002.pdf]

Reducing effects of dispersal on the bias of 2-sample mark-recapture abundance estimators

James N. McNair[1], Carl R. Ruetz III[1], Ariana Carlson[1], and Jiyeon Suh[2]

[1]Annis Water Resources Institute, Grand Valley State University, 740 West Shoreline Drive, Muskegon, Michigan 49441 and [2]Department of Mathematics, Grand Valley State University, 1 Campus Drive, Allendale, Michigan 49401, USA

Supporting information 2: Simulation results

Guide to the histograms and annotations

The 21 figures on the following pages display histograms for 336 cases included in the simulation study. Each figure includes histograms for 16 combinations of capture probabilities (4 choices, each a separate column of histograms in each figure) and maximum movement distance (4 choices, each a separate row of histograms in each figure). The figures are organized as follows:

Figs. 1–3:	Standard sampling scheme, average inward and outward dispersal approximately equal, capture probabilities for samples 1 and 2 equal; true study-area abundance $N = 75$ (Fig. 1), $N = 300$ (Fig. 2), $N = 600$ (Fig. 3)
Figs. 4–6:	Modified sampling scheme, average inward and outward dispersal approximately equal, capture probabilities for samples 1 and 2 equal; $N = 75, 300, 600$
Figs. 7–9:	Modified sampling scheme, average inward and outward dispersal approximately equal, capture probability for sample 1 increased to make sampling effort the same as for sample 2; $N = 75, 300, 600$
Figs. 10–12:	Same as Figs. 1–3 except average inward dispersal is greater than average outward dispersal; $N = 75, 300, 600$
Figs. 13–15:	Same as Figs. 1–3 except average inward dispersal is less than average outward dispersal; $N = 75, 300, 600$
Figs. 16–18:	Same as Figs. 7–9 except average inward dispersal is greater than average outward dispersal; $N = 75, 300, 600$
Figs. 19–21:	Same as Figs. 7–9 except average inward dispersal is less than average outward dispersal; $N = 75, 300, 600$.

Each plot in each figure includes annotations in the title and in the plot panel, as well as three reference lines plotted on the histogram. These are described below.

The following annotations appear in each plot title:

- $\bar{b}$: Average relative bias (%) of Chapman's estimator with respect to initial study-reach abundance n in the simulations
- IQR: Interquartile range of Chapman estimates in the simulations.

The following annotations appear in each plot panel:

q: Capture probability for sample 1
q': Capture probability for sample 2
δ: Maximum movement distance
$\bar{I}$: Average number of individuals that entered the study area between samples 1 and 2 in the simulations
$\bar{p}$: Average proportion of individuals in the study area when sample 1 was taken that were not in the study area when sample 2 was taken, standard sampling scheme only (an estimate of p)
$\bar{p}_{\mathrm{U}}$: Average proportion of individuals in zone U of the study reach when sample 1 was taken that were not in the study reach when sample 2 was taken, modified sampling scheme only (an estimate of p_{U})
$\bar{p}_{\mathrm{C}}$: Average proportion of individuals in zone C of the study reach when sample 1 was taken that were not in the study reach when sample 2 was taken, modified sampling scheme only (an estimate of p_{C})
$\bar{p}_{\mathrm{D}}$: Average proportion of individuals in zone D of the study reach when sample 1 was taken that were not in the study reach when sample 2 was taken, modified sampling scheme only (an estimate of p_{D}).

The following reference lines are plotted on each histogram:

Solid red line: True study-area abundance when sample 1 was taken
Dashed red line: Average true study-area abundance when sample 2 was taken
Dashed blue line: Average value of Chapman's estimator in simulations.

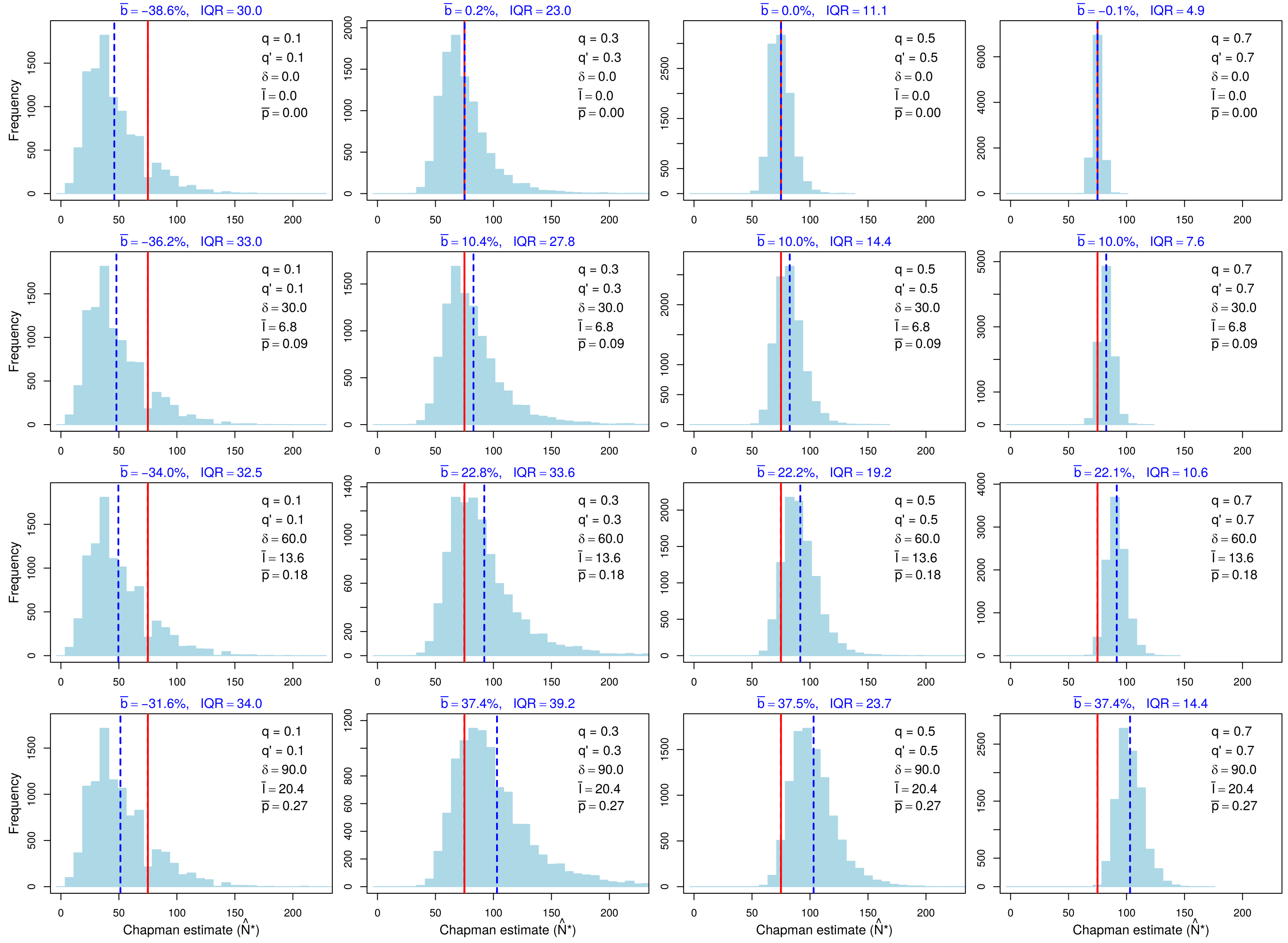

Figure 1. Simulation results with the standard sampling scheme, equal capture probabilities for samples 1 and 2, average inward and outward dispersal approximately equal, and $N = 75$.

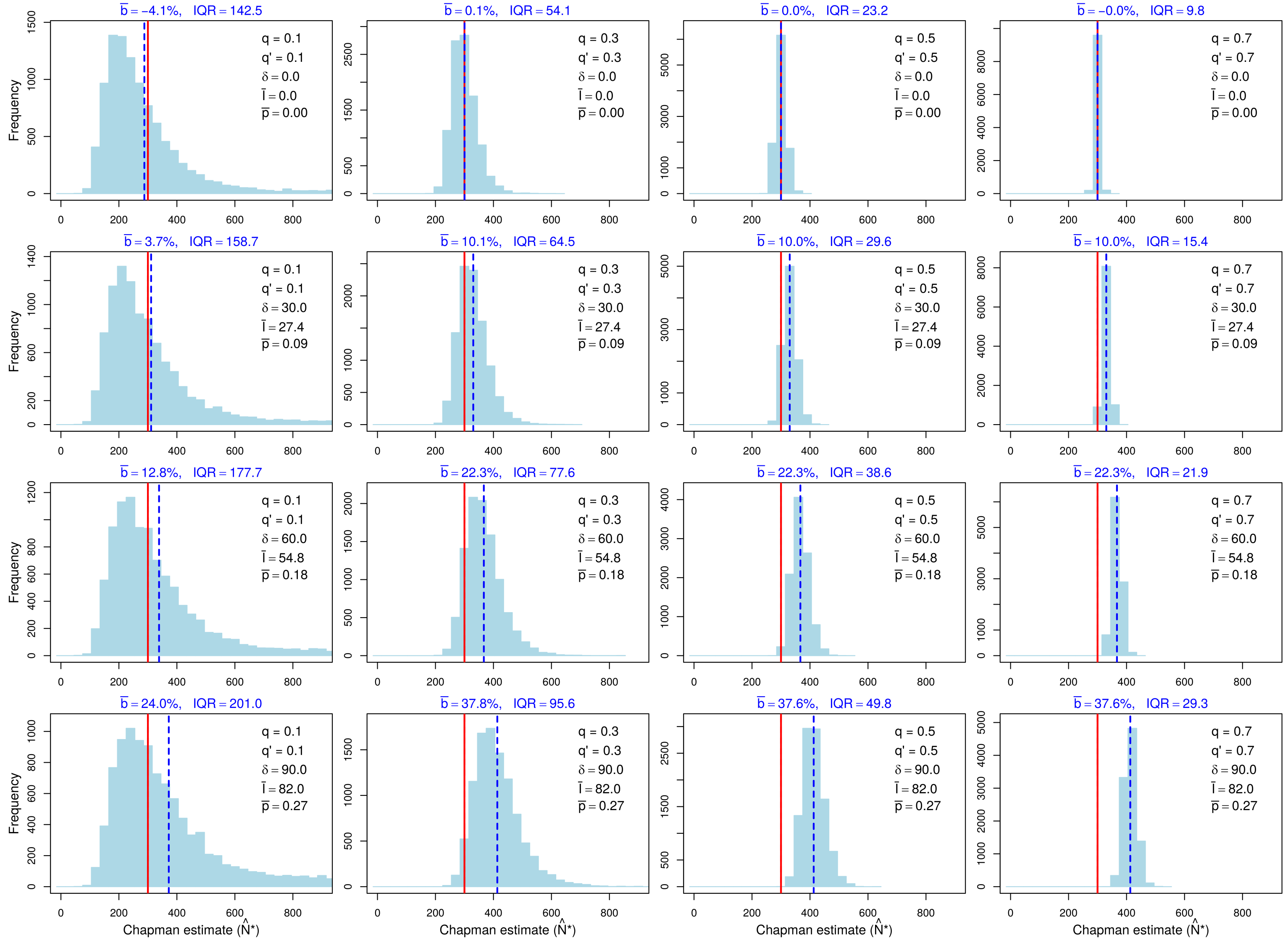

Figure 2. Simulation results with the standard sampling scheme, equal capture probabilities for samples 1 and 2, average inward and outward dispersal approximately equal, and $N = 300$.

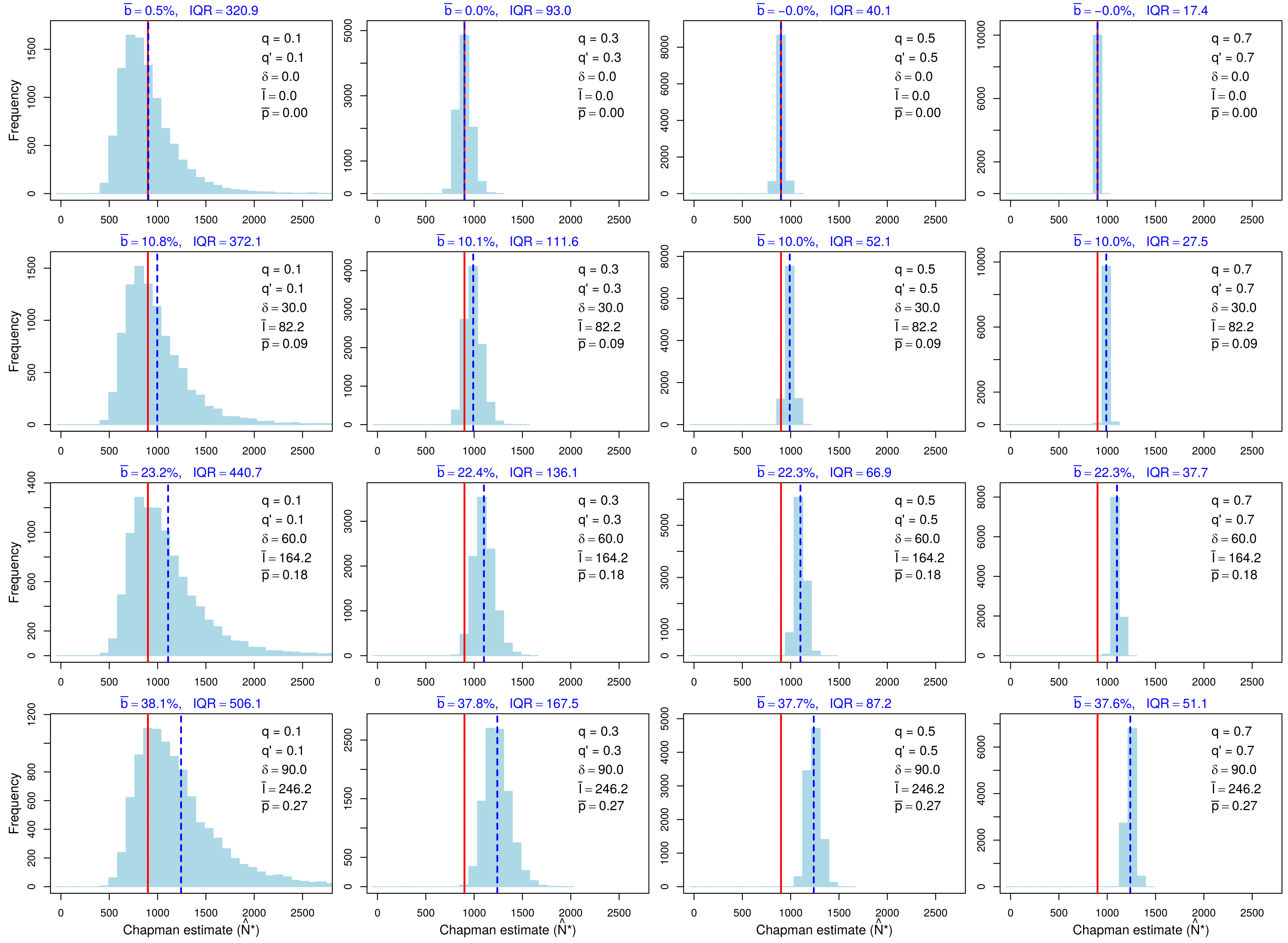

Figure 3. Simulation results with the standard sampling scheme, equal capture probabilities for samples 1 and 2, average inward and outward dispersal approximately equal, and $N = 600$.

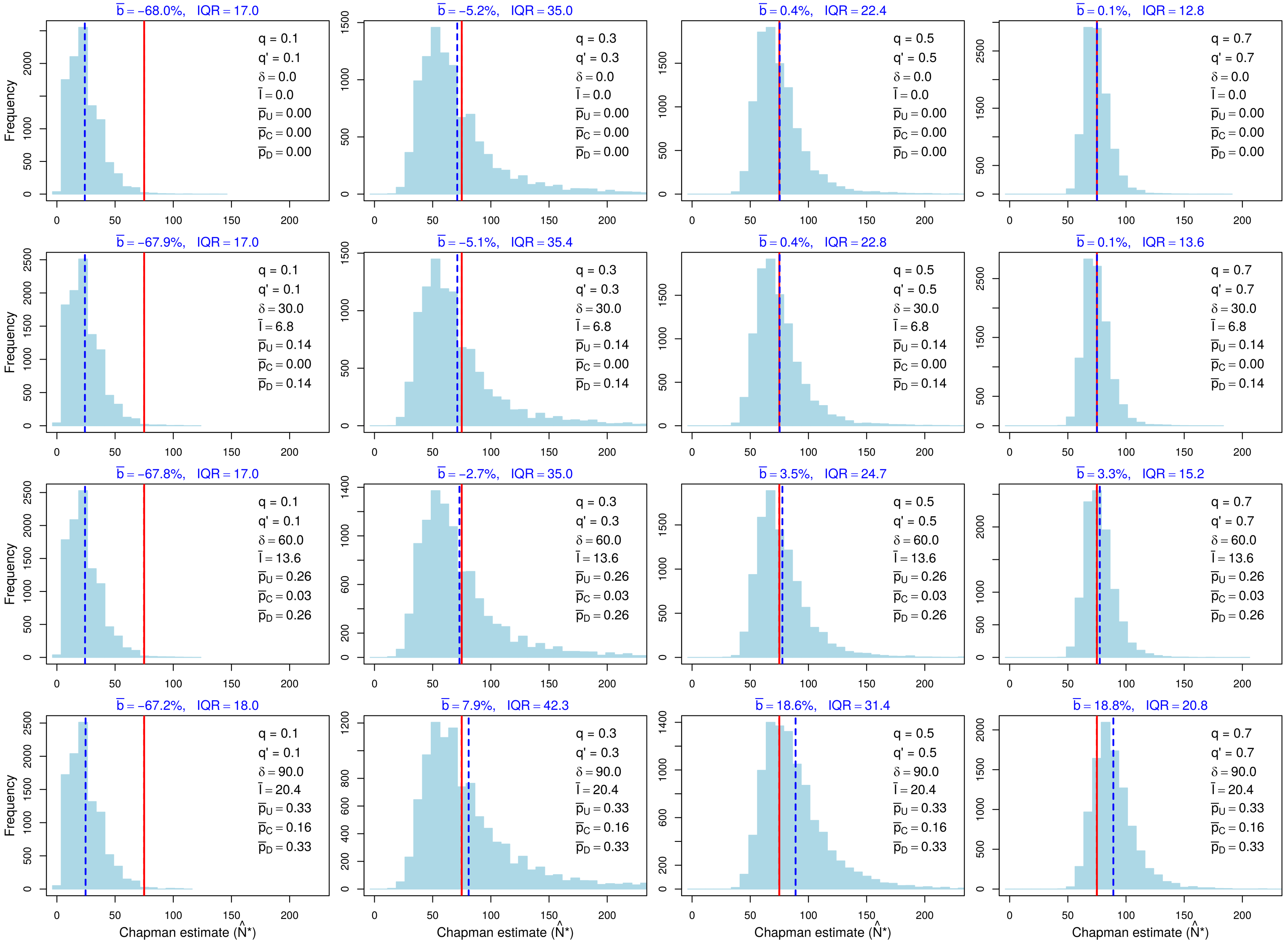

Figure 4. Simulation results with the modified sampling scheme (sample 1 taken from the central zone only), equal capture probabilities for samples 1 and 2 (hence, *sampling effort was lower for sample 1 than for sample 2*), average inward and outward dispersal approximately equal, and $N = 75$.

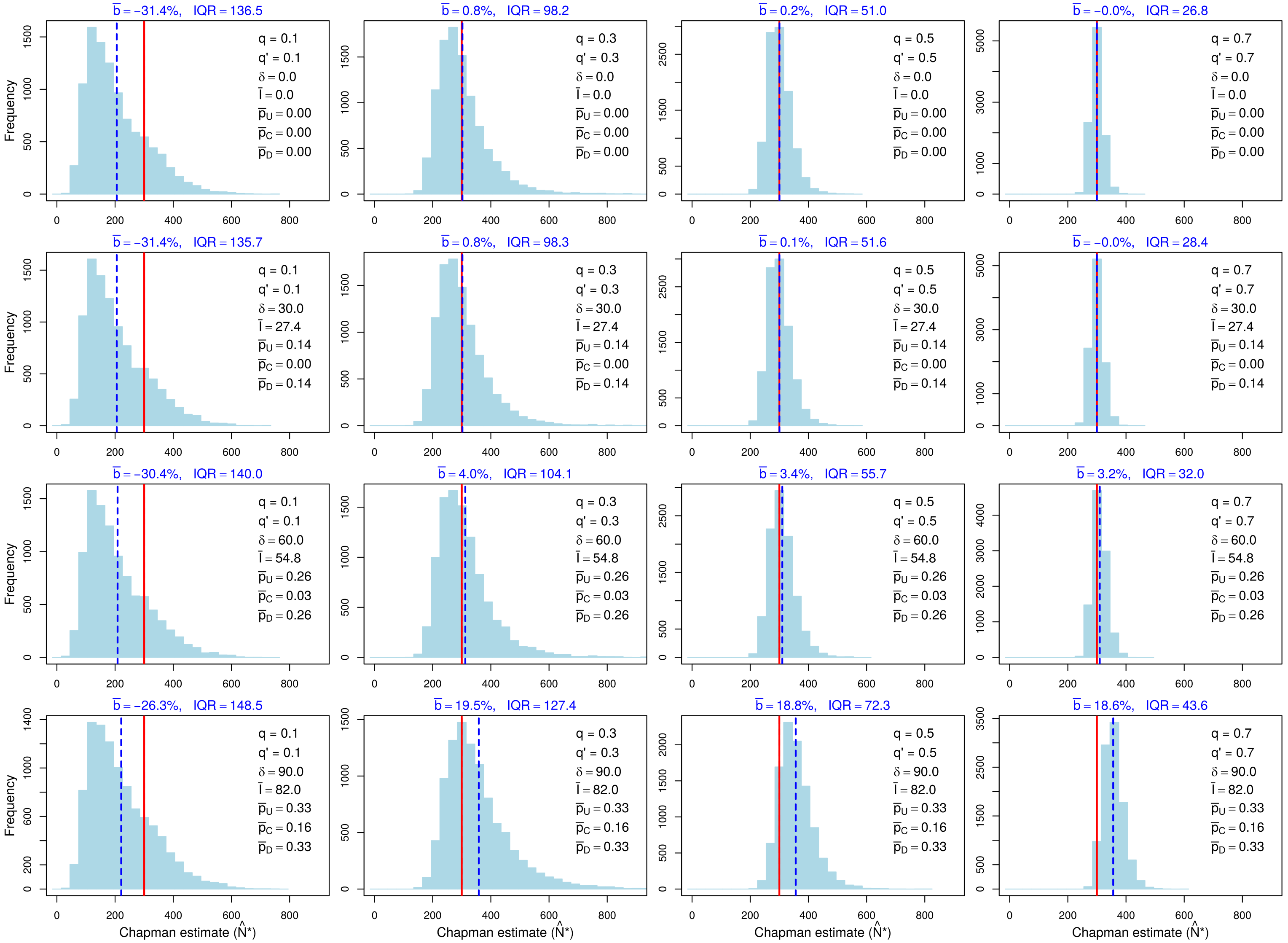

Figure 5. Simulation results with the modified sampling scheme (sample 1 taken from the central zone only), equal capture probabilities for samples 1 and 2 (hence, *sampling effort was lower for sample 1 than for sample 2*), average inward and outward dispersal approximately equal, and $N = 300$.

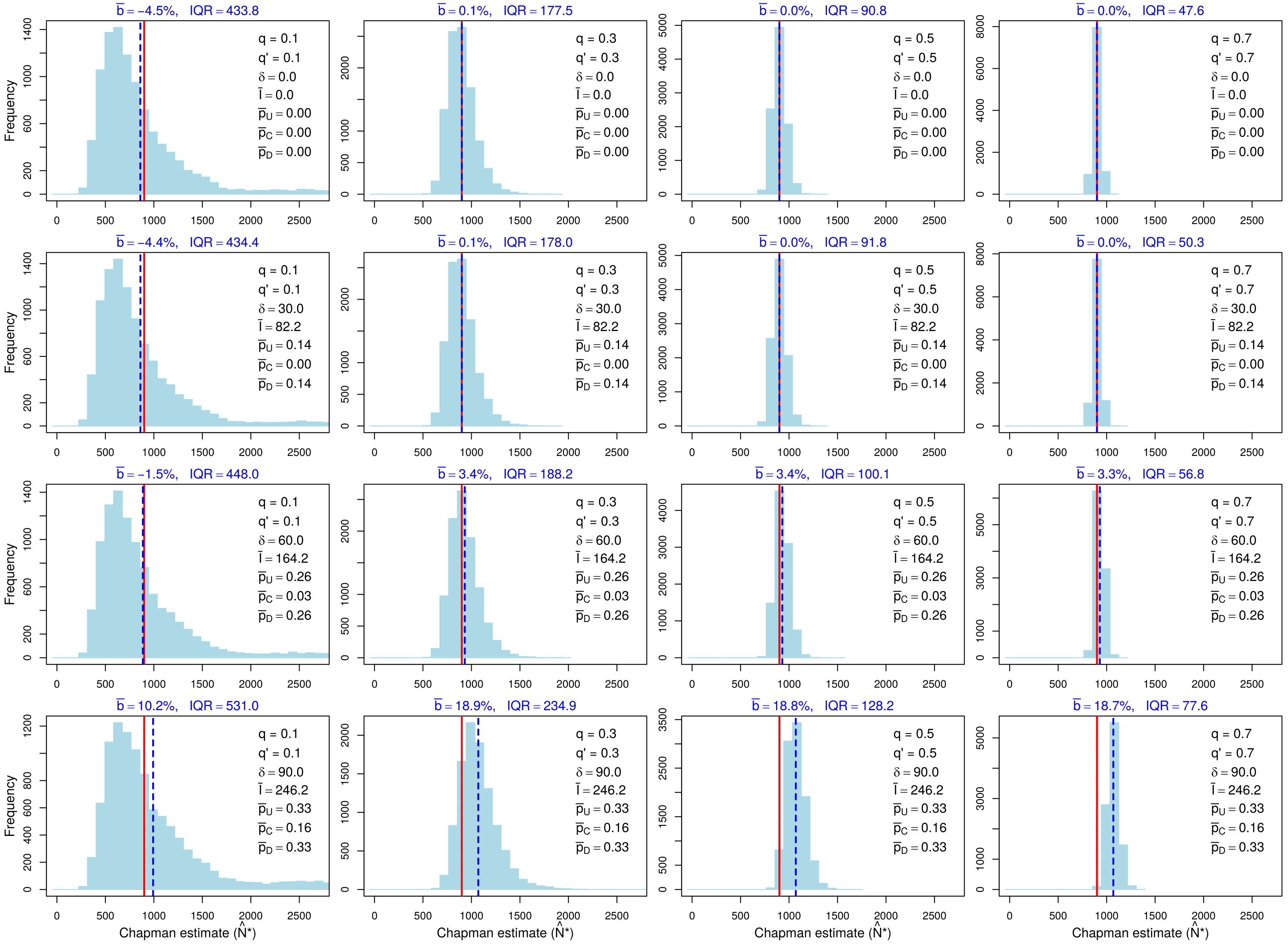

Figure 6. Simulation results with the modified sampling scheme (sample 1 taken from the central zone only), equal capture probabilities for samples 1 and 2 (hence, *sampling effort was lower for sample 1 than for sample 2*), average inward and outward dispersal approximately equal, and $N = 600$.

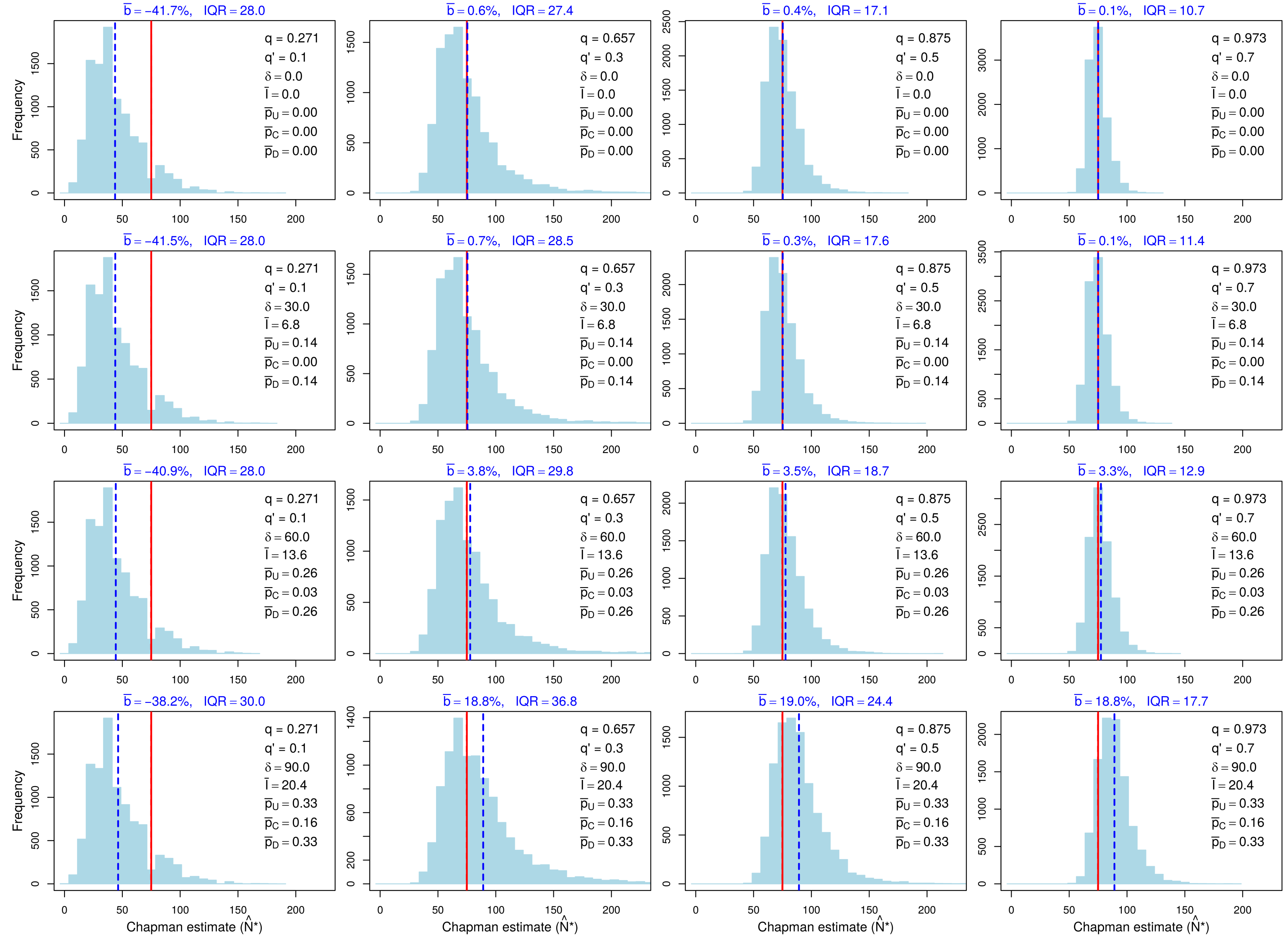

Figure 7. Simulation results with the modified sampling scheme (sample 1 taken from the central zone only), average inward and outward dispersal approximately equal, and $N = 75$. The capture probability for sample 1 was increased using Eq (11) of the text so *sampling effort was the same for samples 1 and 2*.

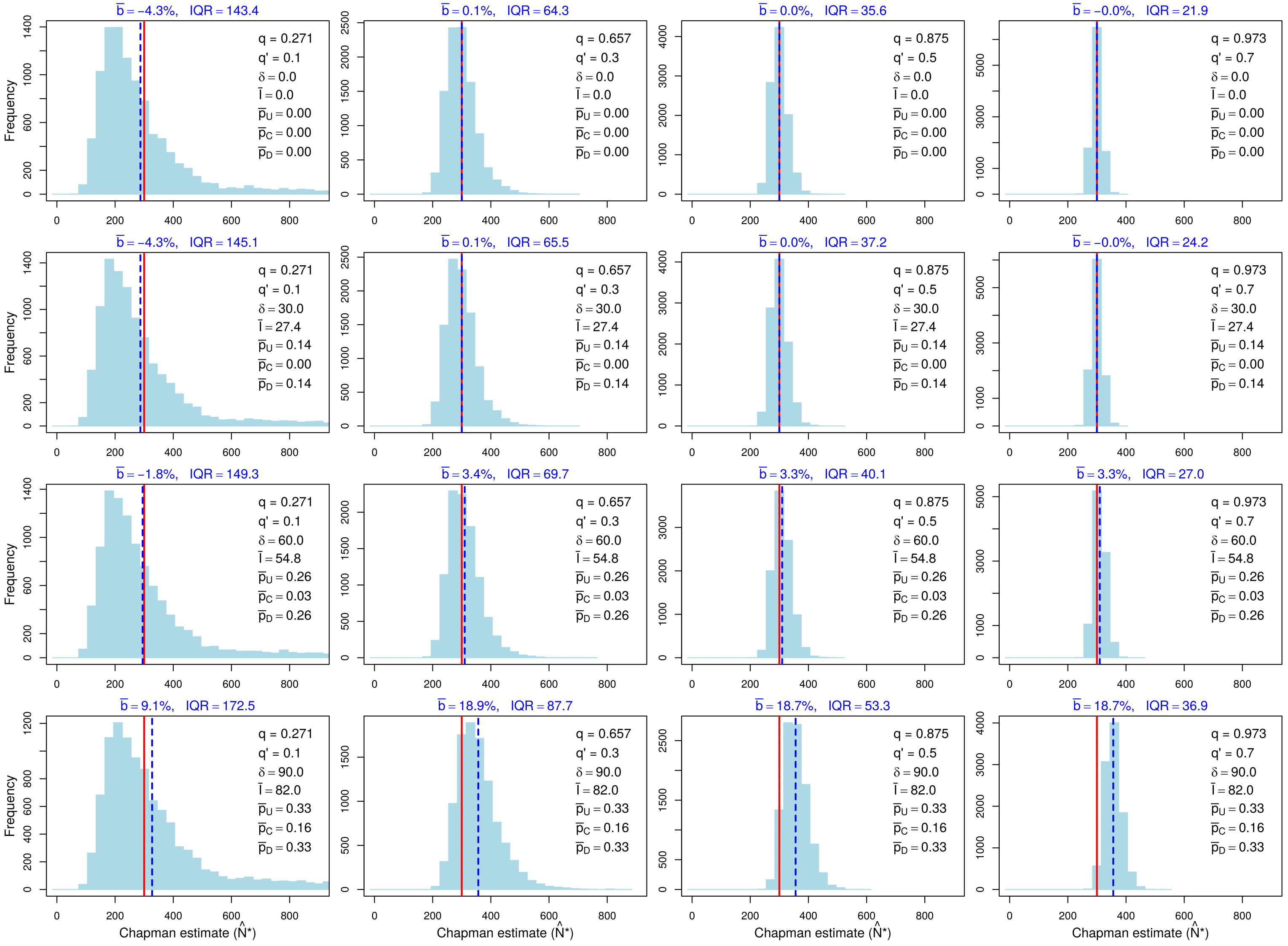

Figure 8. Simulation results with the modified sampling scheme (sample 1 taken from the central zone only), average inward and outward dispersal approximately equal, and N = 300. The capture probability for sample 1 was increased using Eq (11) of the text so *sampling effort was the same for samples 1 and 2*.

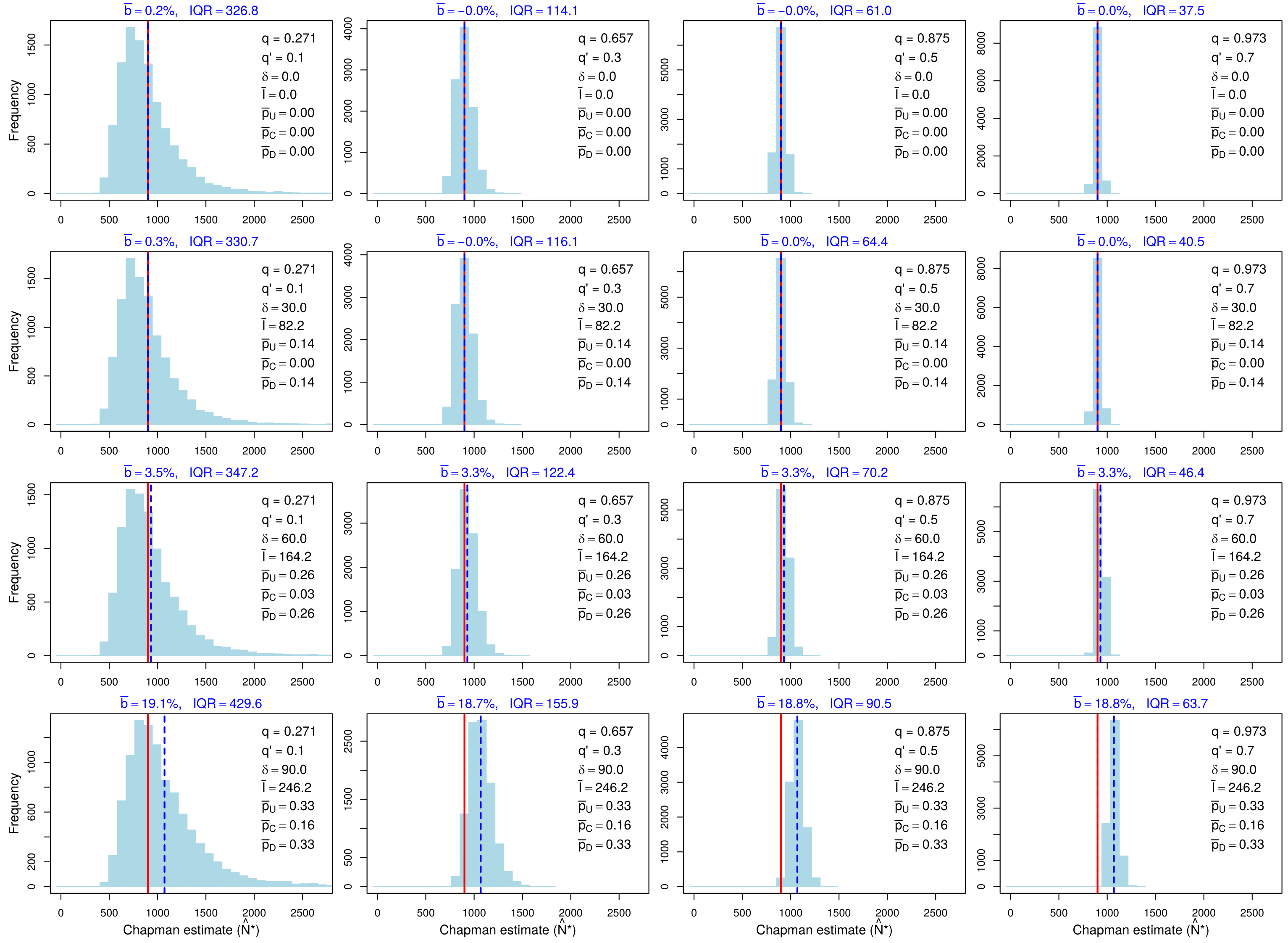

Figure 9. Simulation results with the modified sampling scheme (sample 1 taken from the central zone only), average inward and outward dispersal approximately equal, and N = 600. The capture probability for sample 1 was increased using Eq (11) of the text so *sampling effort was the same for samples 1 and 2*.

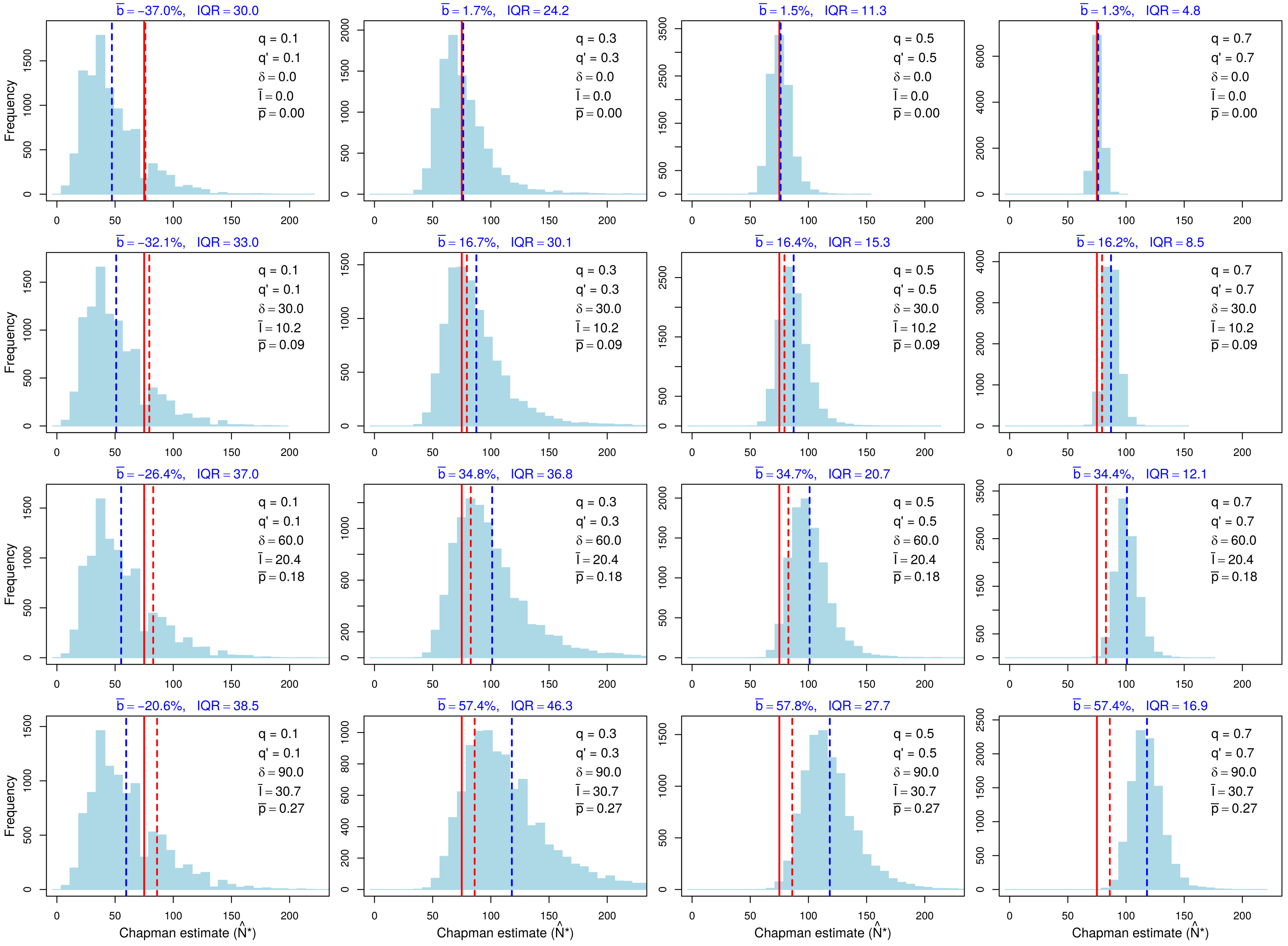

Figure 10. Simulation results with the standard sampling scheme, equal capture probabilities for samples 1 and 2, average inward dispersal greater than average outward dispersal, and $N = 75$.

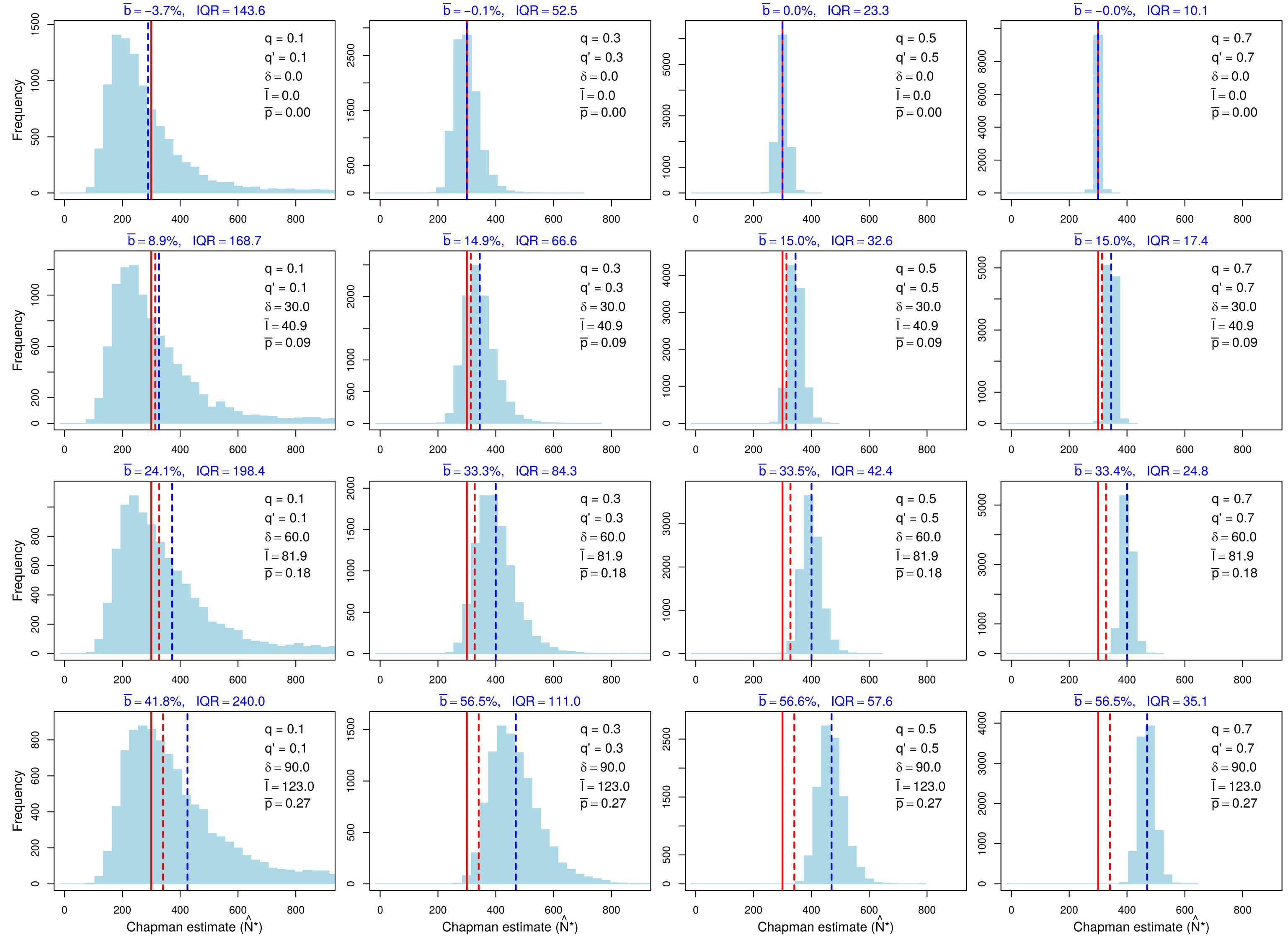

Figure 11. Simulation results with the standard sampling scheme, equal capture probabilities for samples 1 and 2, average inward dispersal greater than average outward dispersal, and N = 300.

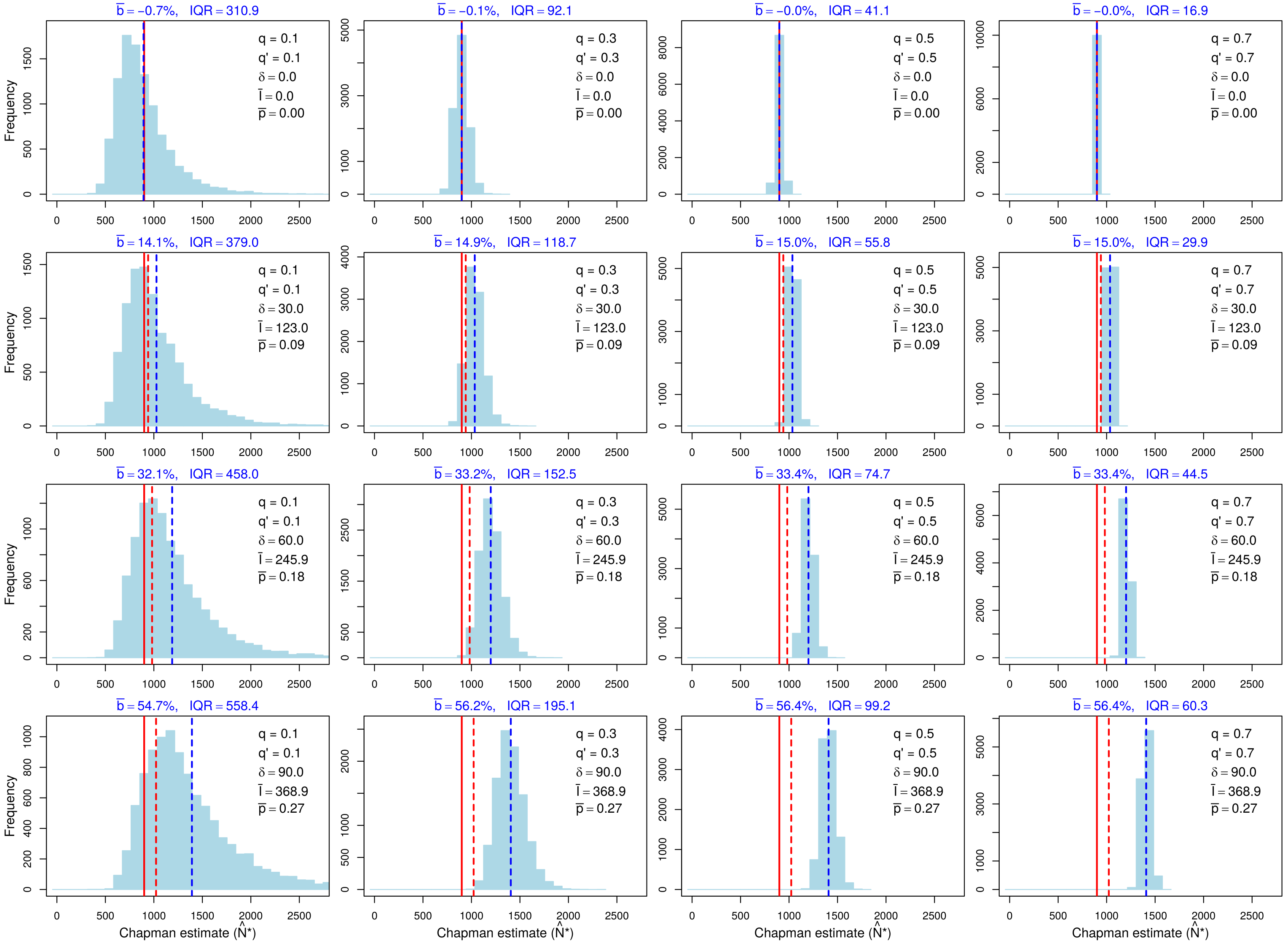

Figure 12. Simulation results with the standard sampling scheme, equal capture probabilities for samples 1 and 2, average inward dispersal greater than average outward dispersal, and N = 600.

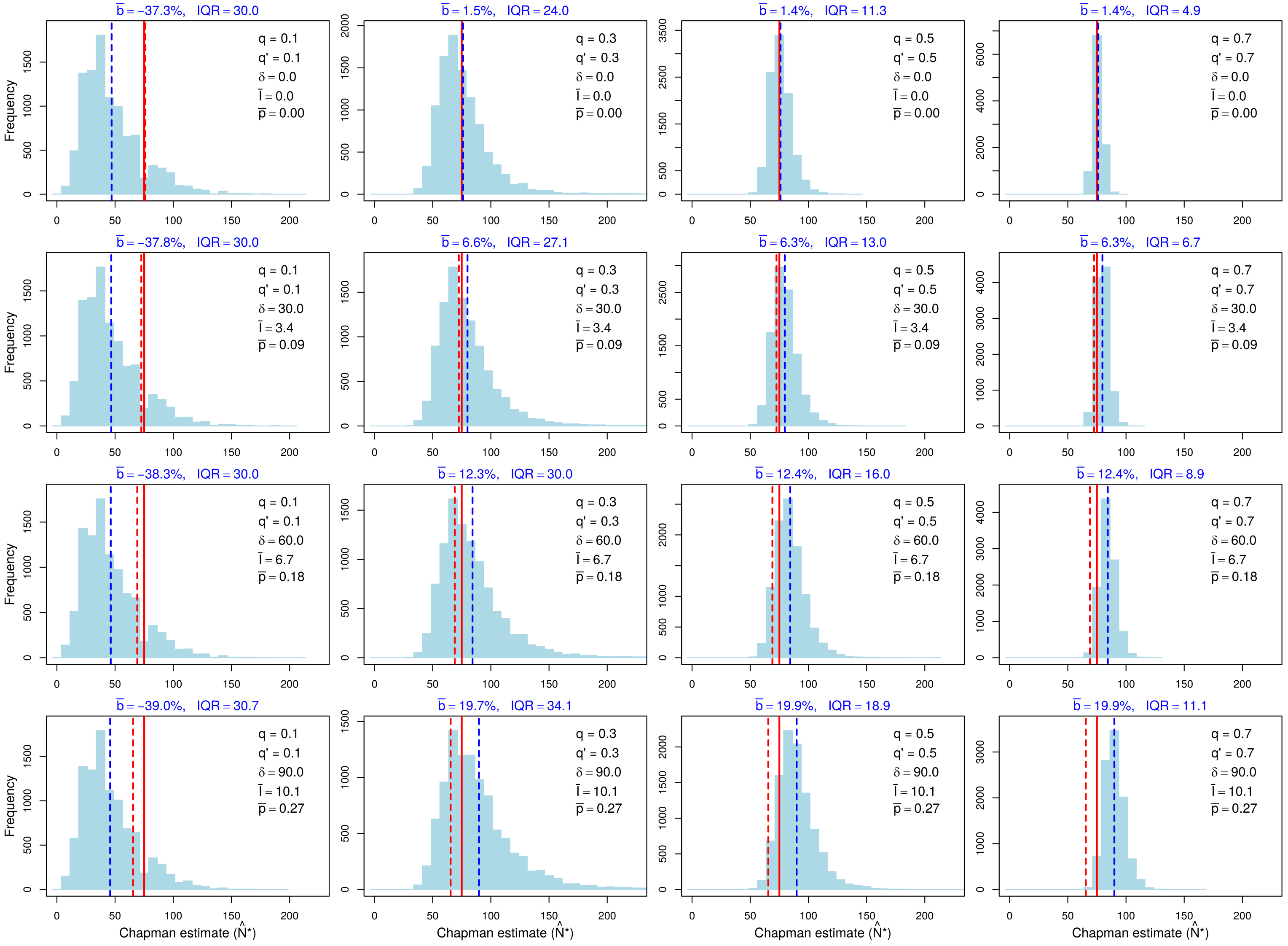

Figure 13. Simulation results with the standard sampling scheme, equal capture probabilities for samples 1 and 2, average inward dispersal less than average outward dispersal, and N = 75.

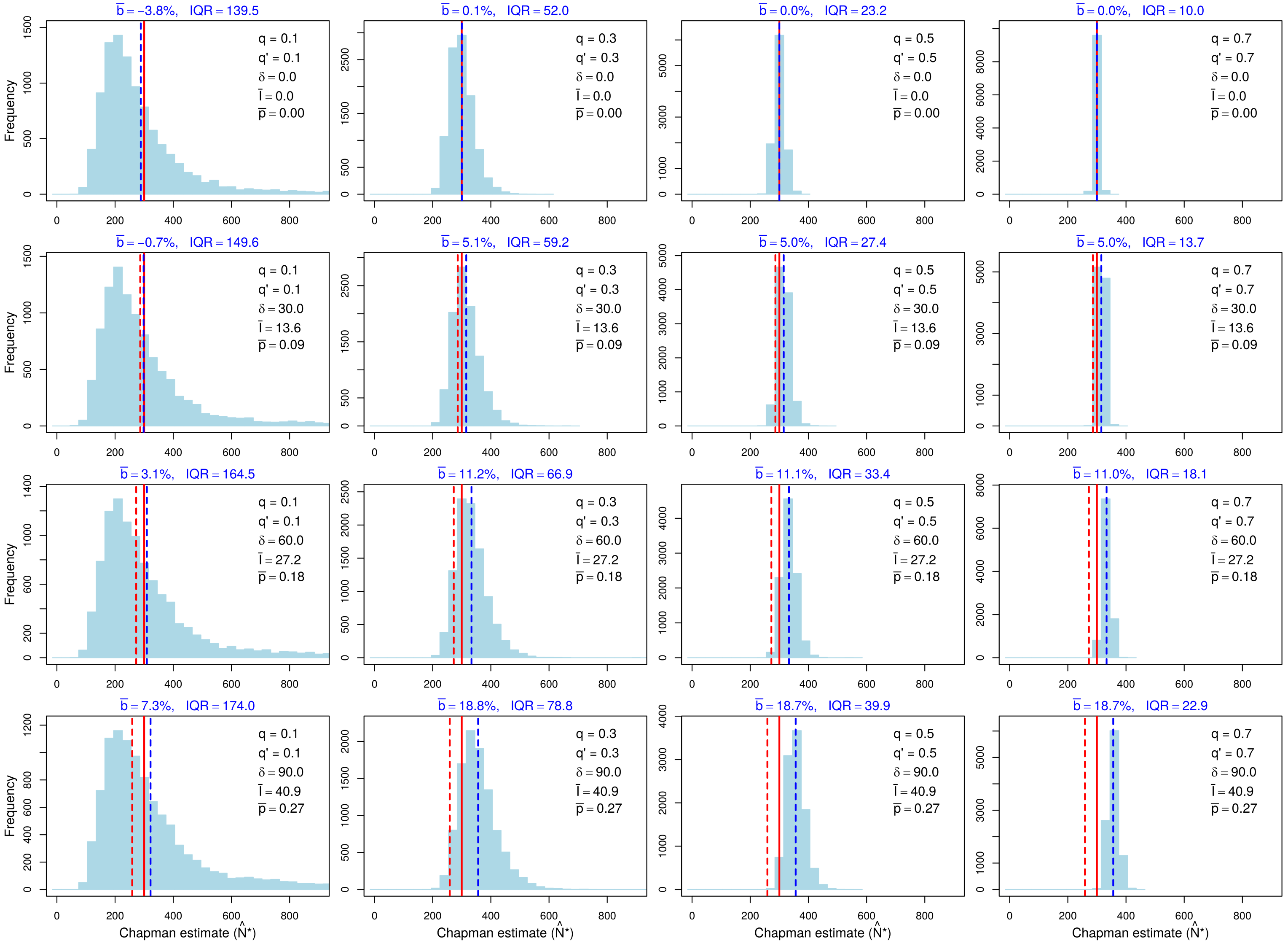

Figure 14. Simulation results with the standard sampling scheme, equal capture probabilities for samples 1 and 2, average inward dispersal less than average outward dispersal, and N = 300.

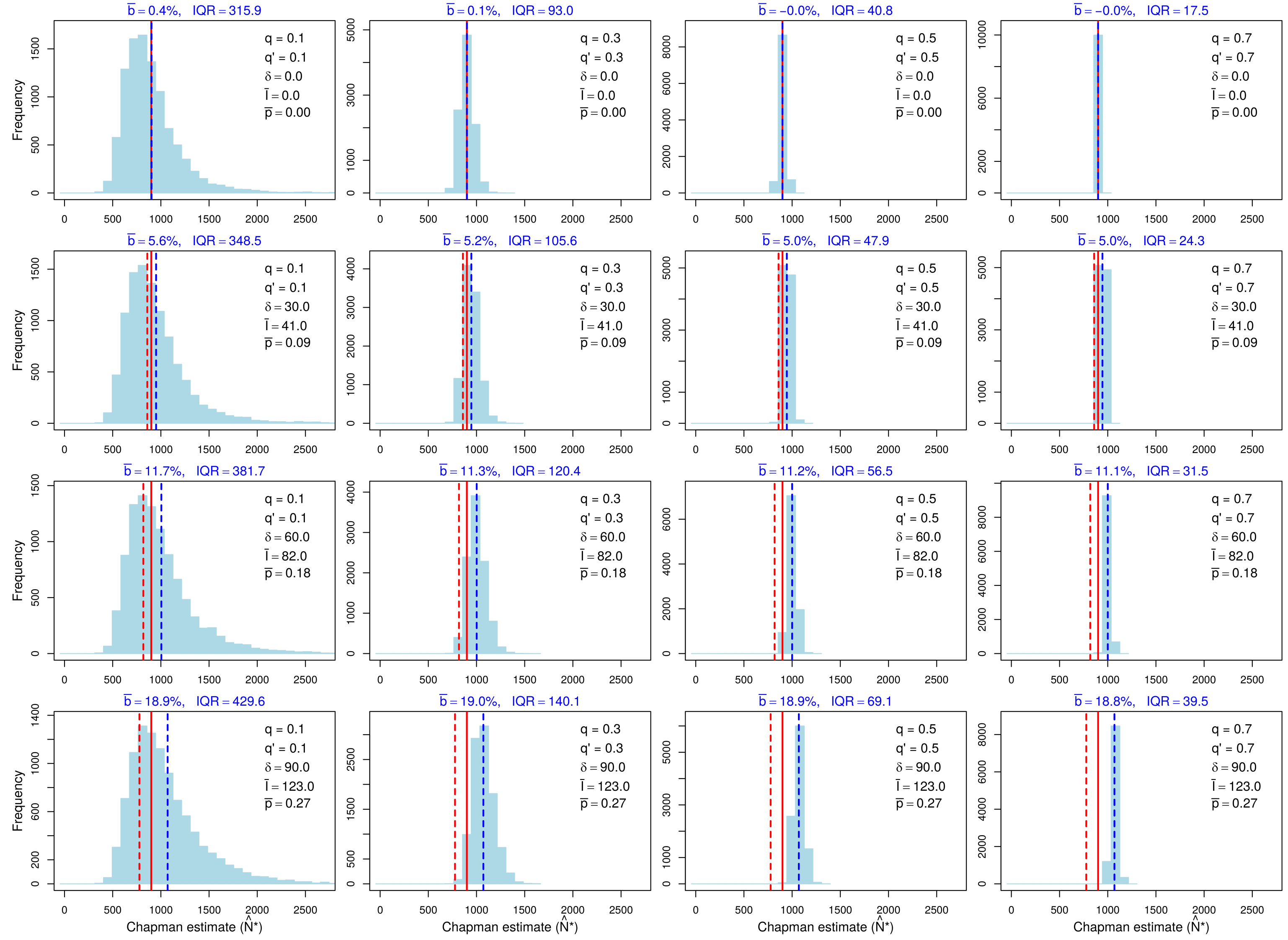

Figure 15. Simulation results with the standard sampling scheme, equal capture probabilities for samples 1 and 2, average inward dispersal less than average outward dispersal, and N = 600.

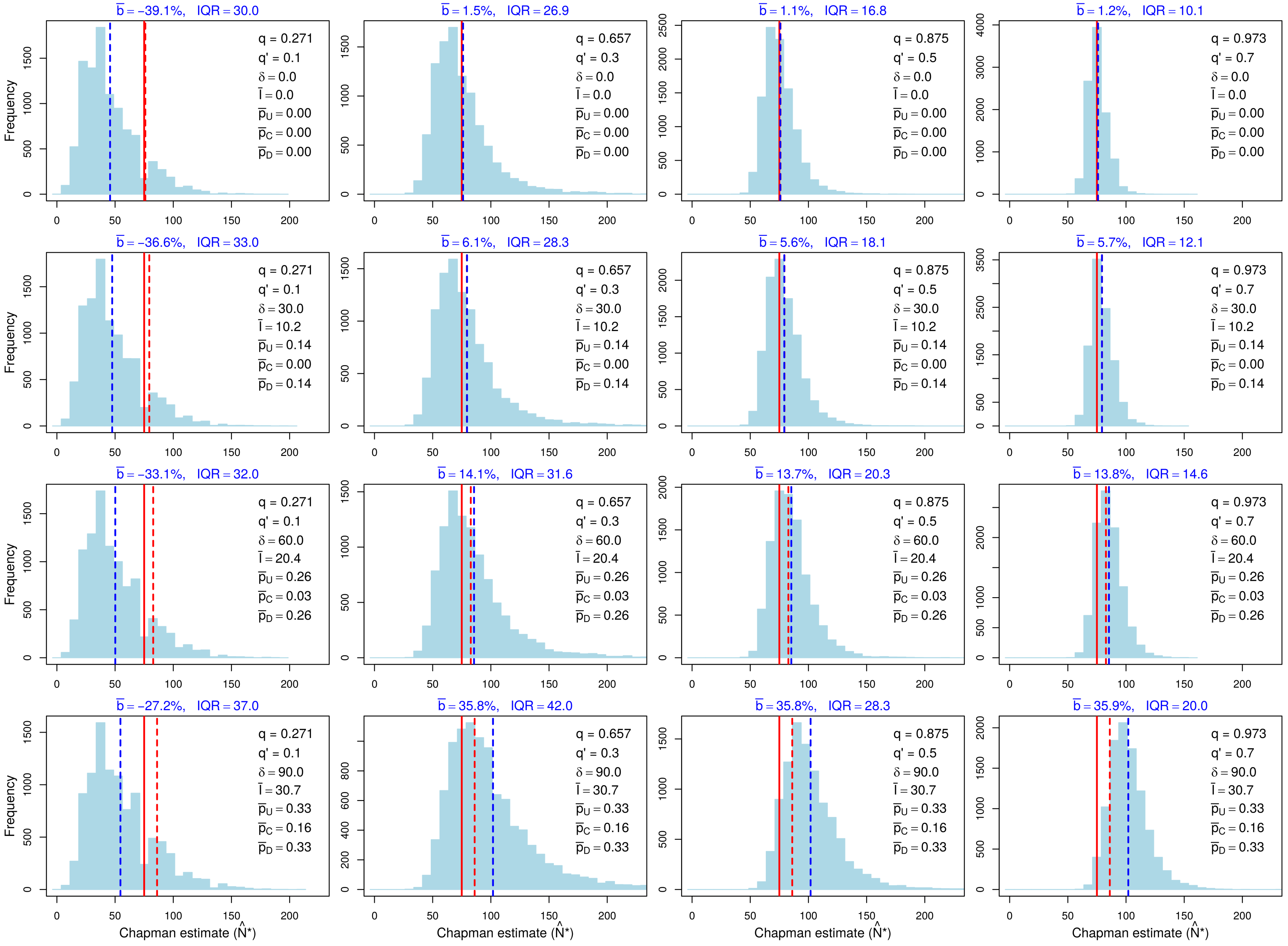

Figure 16. Simulation results with the modified sampling scheme (sample 1 taken from the central zone only), average inward dispersal greater than average outward dispersal, and N = 75. The capture probability for sample 1 was increased using Eq (11) of the text so sampling effort was the same for samples 1 and 2.

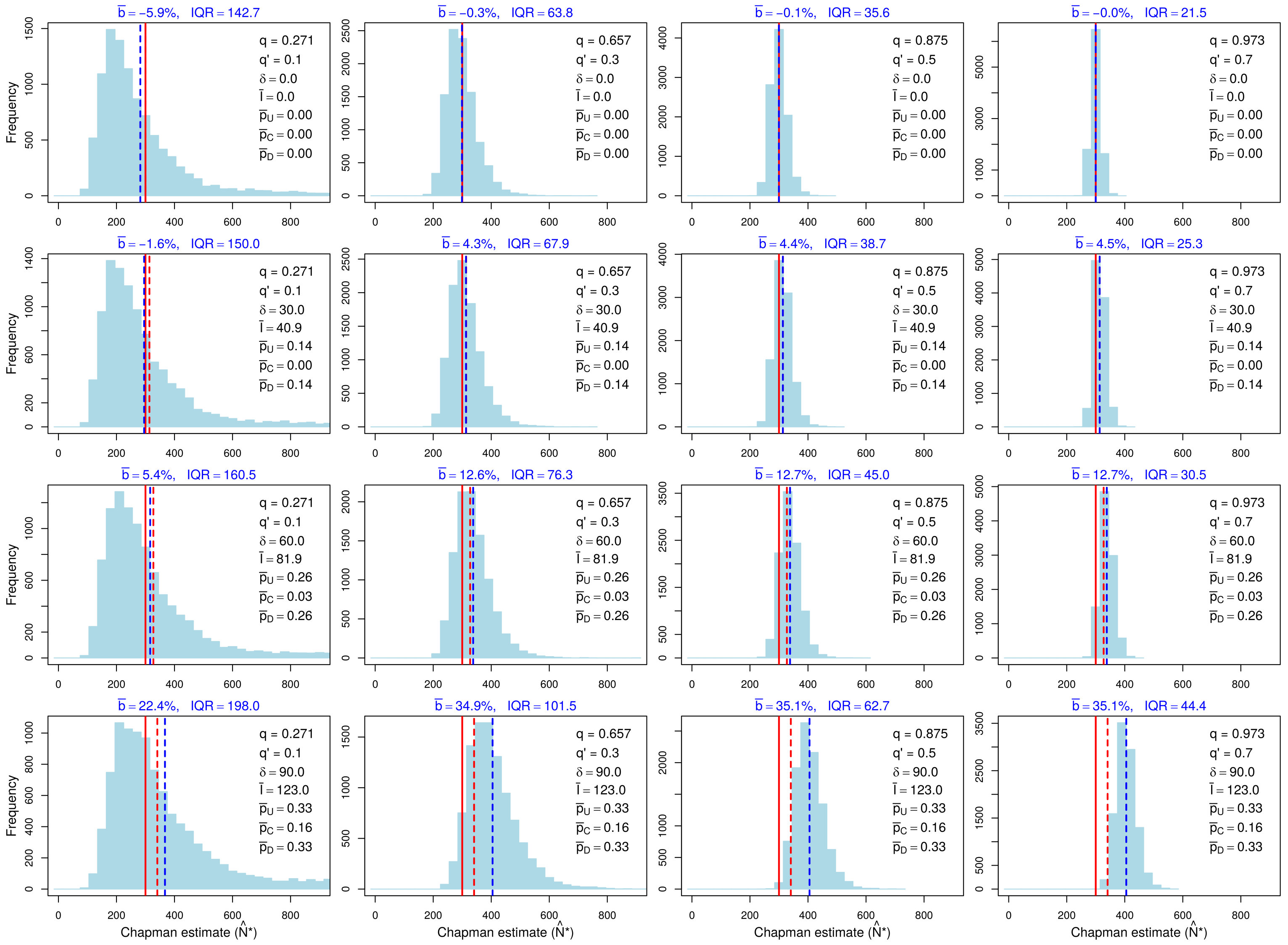

Figure 17. Simulation results with the modified sampling scheme (sample 1 taken from the central zone only), average inward dispersal greater than average outward dispersal, and N = 300. The capture probability for sample 1 was increased using Eq (11) of the text so sampling effort was the same for samples 1 and 2.

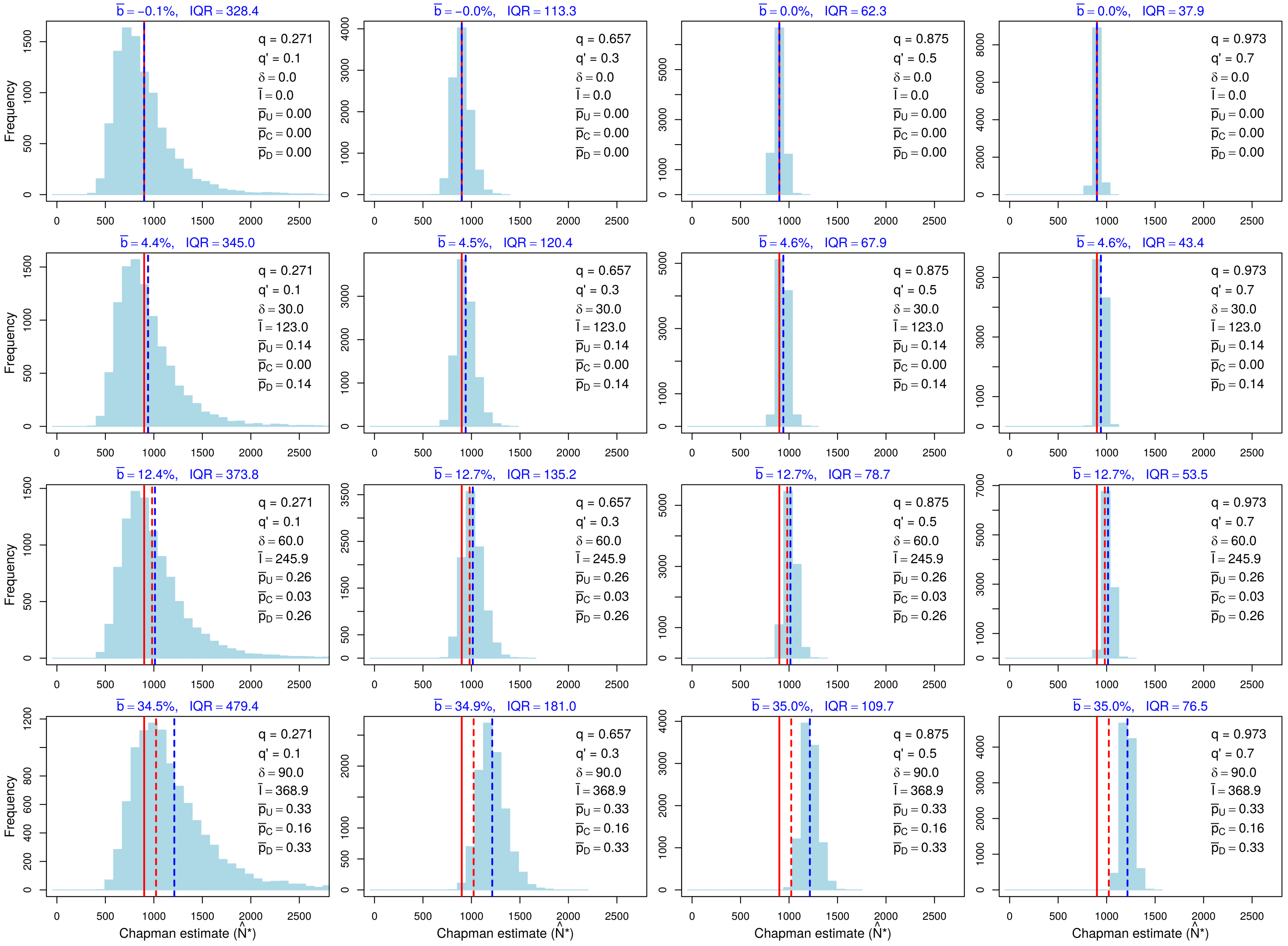

Figure 18. Simulation results with the modified sampling scheme (sample 1 taken from the central zone only), average inward dispersal greater than average outward dispersal, and N = 600. The capture probability for sample 1 was increased using Eq (11) of the text so sampling effort was the same for samples 1 and 2.

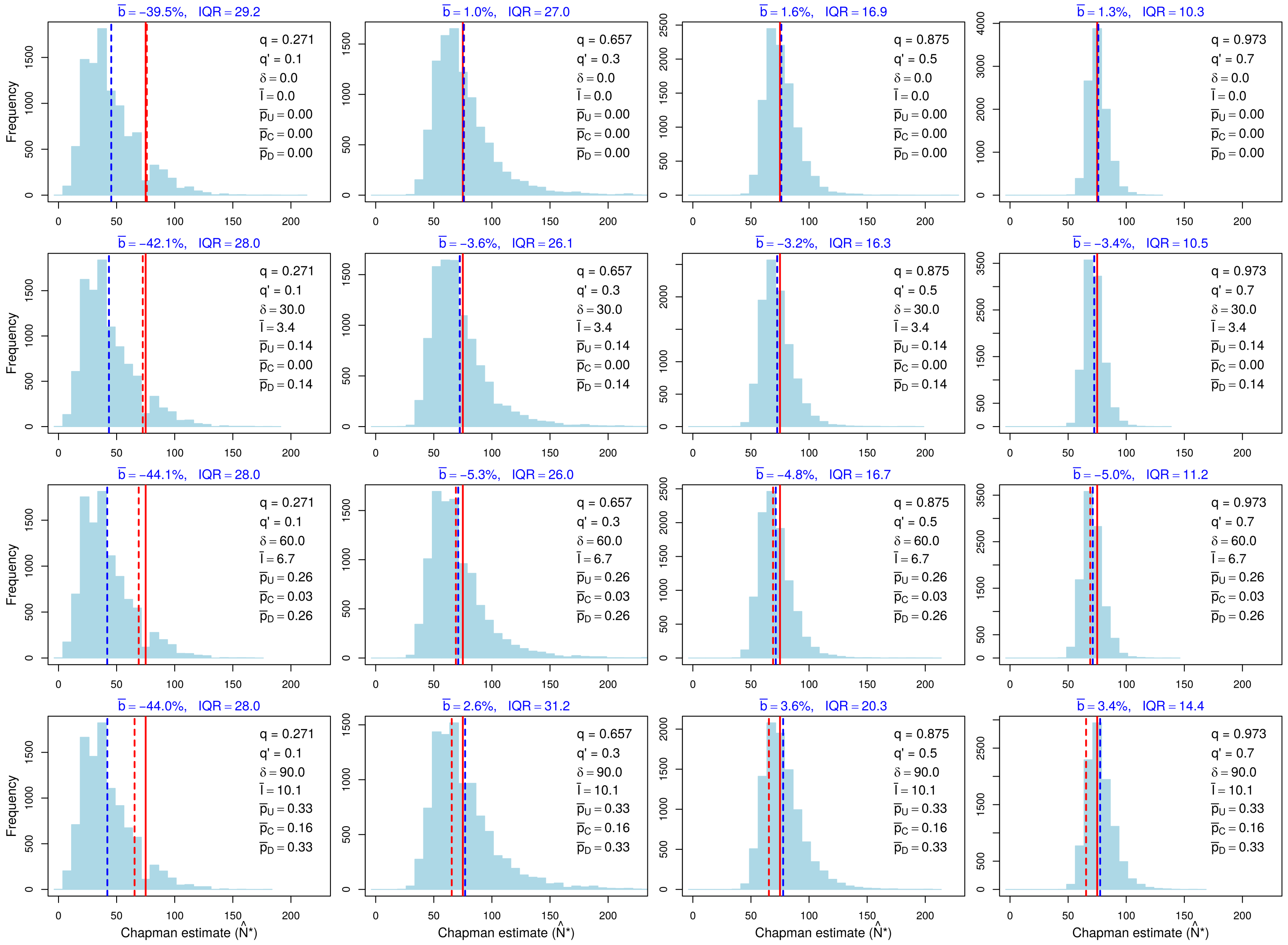

Figure 19. Simulation results with the modified sampling scheme (sample 1 taken from the central zone only), average inward dispersal less than average outward dispersal, and N = 75. The capture probability for sample 1 was increased using Eq (11) of the text so sampling effort was the same for samples 1 and 2.

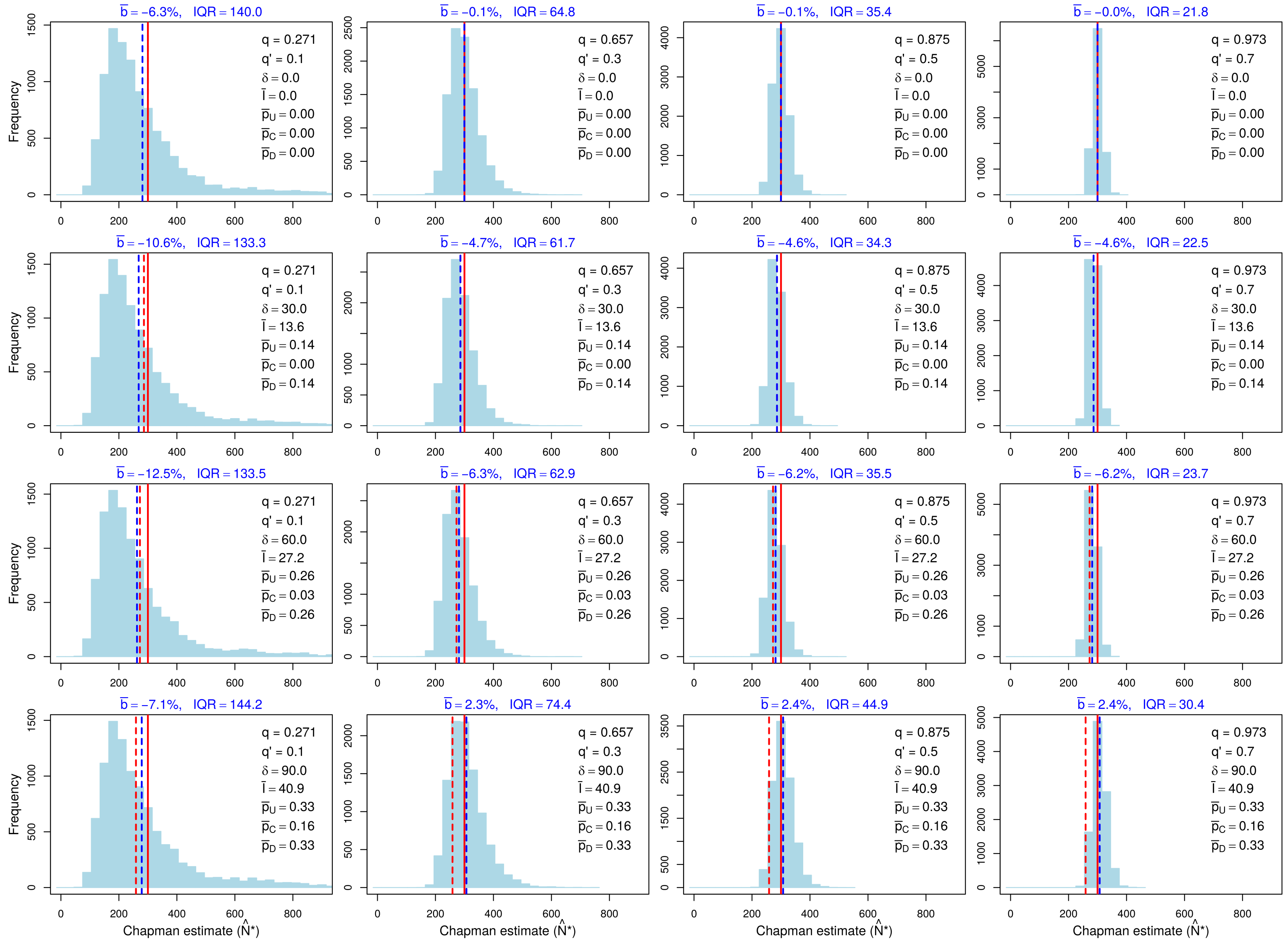

Figure 20. Simulation results with the modified sampling scheme (sample 1 taken from the central zone only), average inward dispersal less than average outward dispersal, and N = 300. The capture probability for sample 1 was increased using Eq (11) of the text so sampling effort was the same for samples 1 and 2.

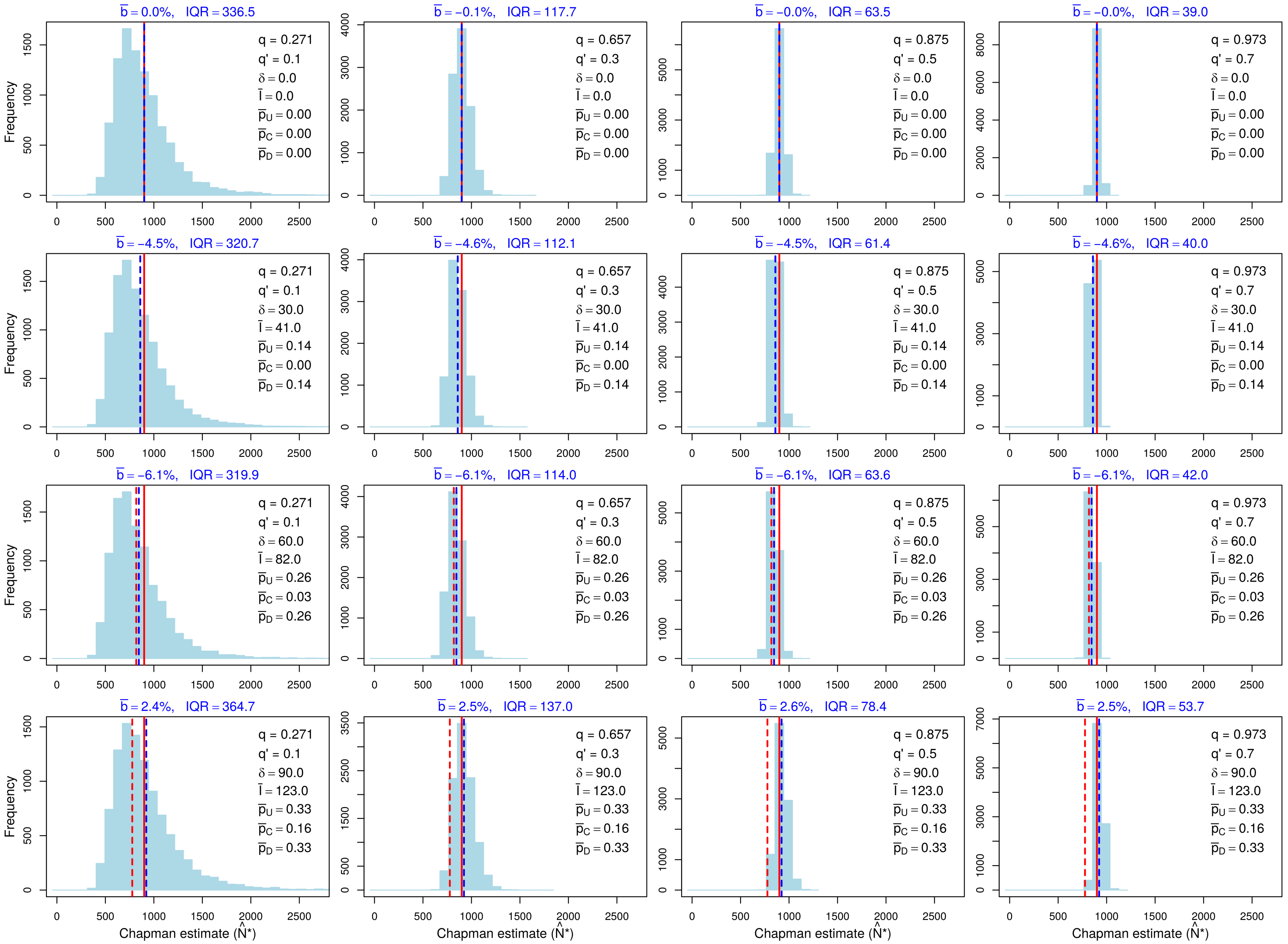

Figure 21. Simulation results with the modified sampling scheme (sample 1 taken from the central zone only), average inward dispersal less than average outward dispersal, and N = 600. The capture probability for sample 1 was increased using Eq (11) of the text so sampling effort was the same for samples 1 and 2.
